# New Advances in Liquid Biopsy Technologies for Anaplastic Lymphoma Kinase (ALK)—Positive Cancer

**DOI:** 10.3390/cancers13205149

**Published:** 2021-10-14

**Authors:** Matteo Villa, Geeta G. Sharma, Chiara Manfroni, Diego Cortinovis, Luca Mologni

**Affiliations:** 1 Department of Medicine and Surgery, University of Milano-Bicocca, 20900 Monza, Italy; m.villa96@campus.unimib.it (M.V.); geeta.geeta@unimib.it (G.G.S.); chiara.manfroni@unimib.it (C.M.); 2 Department of Hematology & Hematopoietic Cell Transplantation, City of Hope National Medical Center, 1500 E Duarte Rd, Duarte, CA 91010, USA; 3 Department of Oncology, San Gerardo Hospital, 20900 Monza, Italy; d.cortinovis@asst-monza.it

**Keywords:** ALK, lung cancer, liquid biopsy

## Abstract

**Simple Summary:**

A new methodology of cancer testing, called “liquid biopsy”, has been under investigation in the past few years. It is based on blood tests that can be analyzed by novel genetics and bioinformatics tools, in order to detect cancer, predict or follow the response to therapies and understand the mechanisms of relapse. This technology is still experimental, yet it has sparked much interest within the scientific community because it promises a new era of cancer management. We here review its application in a subset of tumors characterized by the presence of the *ALK* oncogene: patients affected by these tumors can benefit from targeted therapy, but show frequent relapses, which call for improved methods of disease detection.

**Abstract:**

Cancer cells are characterized by high genetic instability, that favors tumor relapse. The identification of the genetic causes of relapse can direct next-line therapeutic choices. As tumor tissue rebiopsy at disease progression is not always feasible, noninvasive alternative methods are being explored. Liquid biopsy is emerging as a non-invasive, easy and repeatable tool to identify specific molecular alterations and monitor disease response during treatment. The dynamic follow-up provided by this analysis can provide useful predictive information and allow prompt therapeutic actions, tailored to the genetic profile of the recurring disease, several months before radiographic relapse. Oncogenic fusion genes are particularly suited for this type of analysis. Anaplastic Lymphoma Kinase (ALK) is the dominant driver oncogene in several tumors, including Anaplastic Large-Cell Lymphoma (ALCL), Non-Small Cell Lung Cancer (NSCLC) and others. Here we review recent findings in liquid biopsy technologies, including ctDNA, CTCs, exosomes, and other markers that can be investigated from plasma samples, in ALK-positive cancers.

## 1. Introduction

Cancer is a clonal disease characterized by the evolution of heterogeneous subpopulations that follow Darwinian processes of selection. Compared to normal species evolution, tumors show rapid adaptation to the environment, due to their inherent genetic instability and large population size. Next-generation sequencing (NGS) technologies have revolutionized our ability to analyze cancer genetic diversity. From pioneering multi-region sequencing studies to current single-cell analyses, the accumulated data point to high intra-tumor heterogeneity, which poses significant challenges to treatments: tumors continue to evolve under treatment and tend to adapt to a new environment represented by therapies. Under these circumstances, rare clones that are resistant to drugs will emerge due to the evolutionary pressure exerted by the treatment. Genetic evolution can also shape the seeding of distant metastases, through population bottlenecks and the acquisition (selection) of new features that confer the ability to colonize different habitats. It has been shown that metastases can differ significantly from the primary tumor and among them, thus configuring a complex scenario. It has now become clear that personalized molecular portraying of tumors and their clonal architecture, as well as dynamic monitoring of response to treatments, should become a routine procedure in order to optimize the outcome, predict relapses and allow prompt intervention. Although these concepts are rather obvious for most cancers with heterogeneous mutational profiles, they also apply to special cases of tumors driven by a dominant oncogene, such as those harboring oncogenic fusion kinases. In these cases, targeted therapies drive the outgrowth of cells carrying mutations of the target or activation of by-pass signaling pathways.

Anaplastic lymphoma kinase (ALK) is a receptor tyrosine kinase normally expressed primarily on the cell membrane of a specific subset of neurons. Its physiological activity is strictly regulated by ALKALs (ALK And LTK ligands) and by pleiotrophin. Mutant forms of ALK are implicated in a variety of cancers: activating point mutations of the native receptor drive the onset of a subset of neuroblastoma, as well as thyroid, and renal cancer, while oncogenic *ALK* gene translocations or inversions are found in non-small cell lung cancer (NSCLC), anaplastic large-cell lymphoma (ALCL), inflammatory myofibroblastic tumor (IMT) and rare cases of other solid tumors [1]. These rearrangements cause the inadvertent overexpression of a constitutively active form of the kinase, driving aberrant cell survival and uncontrolled proliferation. Knowledge of the precise molecular mechanism of transformation has led to the development of efficacious targeted treatments for ALK-dependent tumors. The introduction of these molecularly targeted drugs has radically changed the prognosis of these patients, demonstrating great efficacy in terms of overall response rate (ORR), progression-free survival (PFS) and overall survival (OS), in particular, compared to chemotherapy. Unfortunately, despite the excellent activity of ALK inhibitors, progression remains inevitable due to the emergence of drug resistance. The mechanisms through which resistance can develop are essentially of three types: amplification of the *ALK* oncogene, activation of alternative signal translation pathways (bypass tracks), and the onset of mutations within the catalytic domain of ALK [2]. The identification of specific resistance mechanisms is of primary importance as it can influence the choice of the next-line therapy.

To obtain information on the genetics of cancer cells, tumor tissue sampling has traditionally been the most widely used approach. Unfortunately, the sample is often inaccessible for biopsy, or qualitatively inadequate for analysis [3]. In particular, recurrent disease sampling is not feasible in many cases. However, as advanced tumors tend to acquire metastatic potential, i.e., the ability to disseminate secondary clones to distant organs through blood circulation, we can interrogate tumor genetics via blood analysis. The so-called liquid biopsy provides a less invasive surrogate method for the identification of somatic mutations through a simple blood draw, without risks to the patient. It is important to note that liquid biopsy represents a sampling from both primary and metastatic sites at the same time, hence it better reflects tumor heterogeneity. Furthermore, as repeated sampling is easily feasible, liquid biopsy constitutes a simple method of real-time longitudinal monitoring during treatment [4], allowing early identification of relapse before clinical manifestation [5]. Here we review current technologies and results obtained by liquid biopsy approaches in ALK-dependent tumors.

## 2. Liquid Biopsy Sources

**Figure 1:**
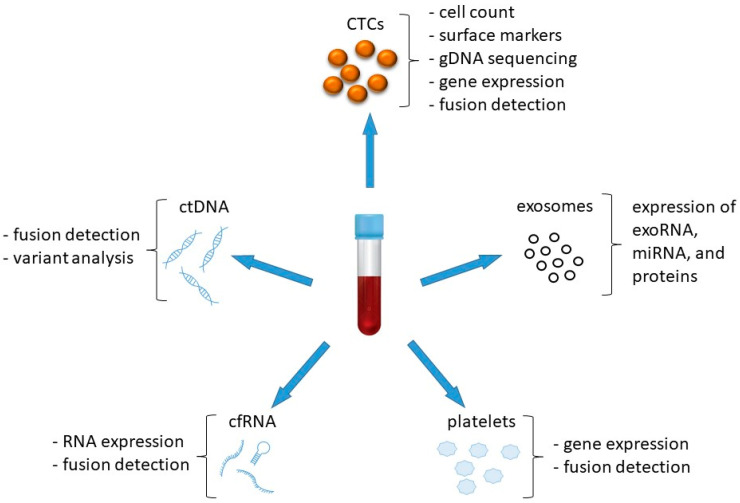
Different sources of biological material can be used for liquid biopsy, starting from a simple blood draw.

Technically, various sources of liquid biopsy material have been described: circulating tumor cells (CTCs), circulating tumor DNA (ctDNA), circulating free RNA (cfRNA), exosomes, platelets (Figure 1). Methods of isolation and analysis have been developed for all four types of analytes, allowing researchers to retrieve from blood several different biomarkers that are representative of the tumor, such as genomic DNA, mRNA, micro-RNA (miRNA), circular RNA (circRNA), proteins and other molecules. CTCs counting, ctDNA concentration, or fusion transcript detection, can help monitor disease burden; ctDNA or CTCs DNA sequencing by NGS technology or droplet digital Polymerase Chain Reaction (ddPCR) can inform on the presence of resistance mutations, either ALK-dependent or by-pass tracks [4,5].

CTCs are cancer cells that break off from the primary tumor mass or from metastases and are shed into the bloodstream [6]. They can be identified by the use of specific surface antigens and their lifespan in the blood is in the range of few hours [7,8]. CTCs from solid tumors have shown prognostic value and can be currently detected by CellSearch^®^, an FDA-approved commercial kit that searches for epithelial cells (CD45−, EpCAM+, cytokeratins 8+, 18+, and 19+) in the blood [9,10,11,12]. While enrichment of CTCs is commonly performed based on the expression of epithelial markers, new strategies independent of epithelial markers have also been developed, such as the use of microfluidics and nanoparticles [13,14,15].

Long before the technology to isolate CTCs became available, circulating cell-free DNA (cfDNA) in blood was reported [16]. cfDNA is defined as the tissue-specific DNA fraction that is released into the bloodstream through various mechanisms such as apoptosis, necrosis and active shedding [17,18]. In comparison to CTCs, cfDNA analysis requires minimal handling as the floating DNA can be easily separated from blood without the need for any special capture technologies. While cfDNA can not be used to analyze cancer morphology or protein expression, it is an equally good source to identify genetic aberrations such as point mutations, genomic rearrangements, gene amplifications or insertion/deletions. The concentration of cfDNA in a healthy subject is approximately 1–100 ng per milliliter of plasma [7]. A clinically relevant fraction of cfDNA (0.01–10%) is represented by circulating tumor DNA (ctDNA), directly released from cancer cells after apoptosis and necrosis [19,20]. The amount of ctDNA varies considerably according to tumor type and stage and its half-life ranges from minutes to few hours [21]. ctDNA offers an excellent noninvasive surrogate biomarker for the detection, as well as longitudinal monitoring, of cancer. With the increased interest in ctDNA as an analyte to detect cancer patients, advancements have been made in improving ctDNA analysis technologies. Various PCR- and NGS-based methods have been developed for the purpose of detecting genetic aberrations in ctDNA for diagnostic purposes [22,23,24,25,26,27,28,29,30,31,32,33].

Similar to ctDNA, miRNAs circulating in blood have the potential to serve as biomarkers for cancer detection [34,35]. miRNAs are a class of 21–25 nucleotide long non-coding RNAs that perform diverse functions, including regulation of their target mRNAs expression [36]. Increasing evidence shows that miRNAs play important roles in tumor biology and regulate the expression of oncogenes and tumor suppressors [37]. The aberrant increase in the expression of some miRNAs can lead to a down-regulation of tumor suppressor genes, while an inadvertent decrease in other miRNAs can lead to the up-regulation of some oncogenes [38,39]. Although RNA is usually considered less stable than DNA, circulating miRNA has shown remarkable stability in blood [40].

Exosomes are a type of extracellular vesicles, ranging from 30 to 120 nm in diameter, that can be released into the extracellular space by eukaryotic cells, including cancer cells. Exosomes carry a variety of genetic materials, such as DNA, mRNA, miRNA, proteins and lipids [41]; in particular, tumor cell-derived exosomes have been shown to cargo specific miRNAs that can be used for liquid diagnostics [42]. Exosomes can bring about changes in cellular processes by acting as messengers and transferring information to the target cells by various mechanisms, such as by fusing with the plasma membrane or by interacting with the protein receptors present on target cells [43]. Exosomes represent an ideal biomarker candidate, as they can be isolated from almost any type of body fluid (blood, urine, cerebrospinal fluid, pleural effusion, etc.) and furthermore, they provide stability and protection from degradation to labile genetic material, such as RNA, thanks to their vesicular structure. They can be isolated by physical, chemical or biological methods based on their size, chemical or biological properties, respectively. For example, EFIRM (electric field-induced release and measurement) is a technique that combines the rapid procedure of extracellular vesicles lysis with subsequent detection and capture of molecular content, thus reducing the degradation caused by exposure [44]. It has been shown that peripheral blood from patients with a malignancy contains higher concentrations of exosomes as compared to healthy individuals. Exosomes derived from cancer patients also carry tumor-specific molecular substances such as genomic DNA with oncogenic mutations and oncoproteins [45,46].

In addition to tumor cells and tumor DNA, normal cells and their components that are present in the tumor microenvironment are also released in the blood. These cells may contain important information about the tumor and thus have the potential to be used as cancer biomarkers. Platelets are an example of such types of cells. In the last few years, several reports have identified an intricate interaction between platelets and cancer cells: tumor-related RNA is released into the blood by several mechanisms; this RNA could function as a communicator between the tumor cells and their microenvironment or distant metastases [47,48,49]. Platelets can internalize circulating tumor-associated RNAs, as well as other biomolecules, becoming so-called “tumor-educated platelets” (TEPs). This makes TEPs a potentially useful tool for cancer diagnostics. Sequencing of mRNA derived from TEPs allowed cancer patients to be differentiated from healthy individuals with an accuracy of 96% [50].

## 3. Liquid Biopsy in ALK+ Cancer

### 3.1. Anaplastic Large-Cell Lymphoma (ALCL)

ALCL is an aggressive peripheral T-cell neoplasm representing 2–3% of all non-Hodgkin lymphomas in adults and 10–15% in the pediatric population [51]. Polychemotherapy is the standard of care for these patients [52]. Despite high response rates, recurrence is observed in around 30% of cases. Although salvage rate is high compared to other T-cell lymphomas, relapsed/refractory patients have a 5-year OS of 50–80% [51,53,54]. ALCL was first found to carry *ALK* rearrangements in 1994, the most frequent being the *NPM/ALK* fusion [53]. Over the last 10 years, the efficacy of ALK inhibitors in this setting has been demonstrated [54,55,56]. Nevertheless, 30–40% of patients treated with ALK inhibitors experience a relapse. The presence of the fusion transcript allows specific detection of rare circulating lymphoma cells. Mussolin et al. showed the prognostic utility of PCR detection of the *NPM/ALK* fusion in the bone marrow (BM) as a marker of minimal disseminated disease (MDD). The authors found that patients with PCR positive BM had a significantly poorer prognosis compared to MDD-negative patients [57]. Another group observed the same correlation and showed that peripheral blood (PB) can also be used for MDD analysis [58]. In another study, pediatric ALCL patients could be stratified into different risk groups by a combination of MDD (from PB or BM) and anti-ALK antibody titre: PFS was 28% for high-risk patients and 93% for the low-risk group [59]. These results were later confirmed in a Japanese study [60]. Detection of minimal residual disease (MRD) by qualitative RT-PCR after the first course of chemotherapy could further divide MDD-positive patients into two subgroups with the different incidence of relapse [61]. More recently, we could amplify by standard RT-PCR the *NPM/ALK* fusion sequence from PB-derived total RNA of patients under crizotinib therapy: deep sequencing of the amplicon allowed the detection of mutations associated with drug resistance [54]. We currently apply this method in clinical routine to identify routes of resistance to ALK inhibitors in ALK+ lymphoma patients, including B-cell cases (Mologni, unpublished data). Along similar lines of research, detection of ALK+/CD30+ CTCs by flow cytometry enabled rapid and cost-effective quantification of MRD in ALCL patients; the results correlated with qPCR data, yet the method showed lower sensitivity compared to PCR [62]. Very recent updates confirmed the prognostic power of MDD/MRD analysis in independent patient cohorts using digital PCR [63] or a standard protocol [64,65]. As an alternative to fusion-specific PCR, Quelen et al. developed a 3′ALK universal amplification protocol, capable to catch all *ALK* fusions, based on the fact that the native gene is not expressed in healthy blood cells; the method showed 100% concordance with standard PCR and the authors proposed it may be applied to liquid biopsy samples [66]. An interesting analysis by Krumbholz and colleagues showed that, in addition to RNA, genomic DNA can be used to track the breakpoint region in NPM/ALK+ ALCL, both from PB and plasma, and use this as an MDD marker [67]. The readers are also referred to an excellent recent review by Mussolin et al. that covers all research on MDD in ALCL [68]. Lastly, exosomes have been investigated for the identification of cancer biomarkers in recent years. In general, exosomes carry a collection of miRNAs that may have a role in disease progression and dissemination. Indeed, several miRNAs have been implicated in ALCL pathobiology, both ALK-positive and ALK-negative [69,70,71,72]. A recent RNA-seq analysis showed that a particular small RNA species was most abundant in circulating exosomes from ALCL patients compared with samples from healthy donors: the large majority of mapped reads derived from the *RNY4* gene, that transcribes a non-miRNA small YRNA involved in mRNA stability and alternative splicing. Furthermore, the *RNY4* load in exosomes of ALCL patients correlated with disease stage. Hence, the authors suggested that exosome-encapsulated *RNY4* might be used as a novel biomarker for ALCL liquid biopsy [73]. A parallel proteomic analysis led to the identification of proteins involved in PI3K signaling that are enriched in exosomes from ALCL patients. The authors proposed three proteins, namely tenascin C, osteopontin and heat shock protein 90 as potential biomarkers for ALCL prognostic stratification [74]. Altogether, these studies open the possibility to assess the risk of relapse and to monitor the response to therapy in a disease where tissue re-biopsies are often difficult to obtain.

### 3.2. Non-Small Cell Lung Cancer (NSCLC)

NSCLC is the most prevalent histological subtype of lung cancer, accounting for approximately 85% of all lung cancer cases worldwide [75]. While surgical resection with or without adjuvant cytotoxic chemotherapy is the mainstay treatment for early-stage NSCLC patients, oncogene-addicted and advanced-stage NSCLC patients are treated with targeted or immunotherapies. Chromosomal rearrangements involving *ALK* were first identified in NSCLC in 2007 where the 3′ region of the *ALK* gene was found fused with the 5′ sequence of the echinoderm microtubule-associated protein-like 4 (*EML4*) gene resulting in the expression of the EML4-ALK oncogenic fusion protein [76,77]. ALK+ NSCLCs are dependent on the activity of the fusion kinase, hence inhibition of ALK leads to the selective elimination of cancer cells. These discoveries led to the development of ALK inhibitor-based treatments [78]. Confirmation of the presence of ALK fusions for diagnostic purposes is usually performed using fluorescence in situ hybridization (FISH) and immunohistochemistry (IHC) of biopsy or surgically resected tissues, the latter considered the gold standard technique [79,80,81,82]. In addition, quantitative PCR has also been used to detect *ALK* transcripts in primary samples [83]. While RT-PCR is one of the simplest and most sensitive techniques to detect *ALK*, the results are heavily dependent on the quality of starting RNA material, which is not very high in formalin-fixed paraffin-embedded (FFPE) specimens. Up to 20% of biopsies are inadequate for molecular testing due to insufficient tissue amounts and re-biopsy at the diagnosis or at relapse is often unfeasible. The lack of sufficient tissue material, as well as difficulties in obtaining tissue from high-risk patients, impelled the development of alternative assays for diagnostic purposes. In such scenarios, liquid biopsy allows for the analysis of several blood-based biomarkers, including the detection of driver oncogenes, enabling molecular diagnosis [84,85].

Despite substantial survival benefits after exposure to first- (crizotinib) or second/third-generation TKIs (ceritinib, alectinib, brigatinib, ensartinib, lorlatinib) all patients acquire resistance to the inhibitor in a relatively short time, while some patients do not respond from the start (primary resistance) [2]. The utility of liquid biopsy in this setting is particularly attractive to identify this cancer at an early stage, select the best treatment option for patients and at the same time monitor the response to treatment, assess the risk of metastasis and prognosis of patients [86,87,88]. In addition, frequent sampling can anticipate the detection of resistance mechanisms [46,89].

#### 3.2.1. Circulating Tumor Cells (CTCs)

Attempts to use CTC detection as a lung cancer biomarker have been made over the last 10 years [90,91,92,93]. In one of the first reports on the detection of ALK rearrangements in CTCs from 34 NSCLC patients [94], 100% concordance was observed between CTCs and tissue biopsies (Table 1). Interestingly, ALK staining in CTCs was more homogenous compared to IHC or FISH from the tumor. In another study, CTCs were isolated and probed for *ALK* using an optimized method named Filter-adapted FISH (FA-FISH). Using a cut-off value of four CTCs, *ALK* detection using CTCs had a sensitivity and specificity of 100% and had a 99.99% correlation with tumor biopsy analysis [95]. The authors also reported that *ALK*-rearranged CTCs mostly showed a mesenchymal phenotype and a distinct split pattern for *ALK* rearrangement suggesting the clonal selection of CTCs with superior migratory and invasive properties. Tan and colleagues reported similar results including a high concordance (~90%) of *ALK* rearrangement detection between CTCs and tumor tissue, higher vimentin expression in CTCs compared to the primary tumor (indicative of an epithelial-to-mesenchymal transition [EMT] phenotype) and a cut-off of four CTCs in ALK-positive samples [96]. The use of 3D imaging for the detection of ALK fusion in CTCs was tested in a small cohort of lung cancer patients: comparing subjects with ALK-positive and ALK-negative NSCLC, the assay was able to capture a good probes signal separation, indicative of *ALK* translocations, by nuclear volume imaging. The authors proposed that the use of 3D DNA FISH could be applied in the routine determination of *ALK* translocations in NSCLC liquid biopsies [97]. Recent results from the prospective multicenter STALKLUNG01 trial validated the clinical feasibility of *ALK* rearrangement detection in CTCs, particularly by immunochemistry [98]. However, no association of CTC counts with OS or PFS was found.

While baseline detection of ALK rearrangement in CTCs does not necessarily predict PFS, the presence of EML4/ALK+ CTCs with *ALK* copy number gain after TKI treatment is associated with poor PFS, therefore it is a signal of drug resistance [112,113]. A recent investigation of 6 ALK inhibitor-resistant patients confirmed the utility of CTC copy number analysis: all CTCs isolated from peripheral blood showed highly aberrant CNA profiles, including *ALK* gain in all cells from one patient, as well as high chromosomal instability; moreover, non-epithelial ALK+ cells were found, suggesting EMT [114]. A report of two EML4-ALK+ NSCLC cases under TKI further highlighted the predictive value of CTC liquid biopsy, which allowed to differentiate the two patients in their clinical course: re-emergence of CTCs during follow-up correlated with disease progression [115]. Finally, while CTC count can have prognostic value in ALK+ NSCLC, CTCs can also be used to shed light on drug resistance mechanisms: an L1196M mutation (Table 2) was found by CTC gene sequencing in the peripheral blood of patients with acquired resistance to crizotinib [116]. Pailler et al. detected drug-resistant mutations in CTCs from 17 patients progressed on crizotinib (*n* = 14) and lorlatinib (*n* = 3). Interestingly, one lorlatinib-resistant patient showed two different compound ALK mutations in different CTCs, sharing the refractory G1202R substitution [117].

#### 3.2.2. Circulating DNA

ctDNA detection in earlier studies relied on the use of allele-specific PCR [27]. However, the method suffered from several limitations such as the limit of detection and required prior knowledge of the specific mutations. In this regard, gene fusions are more easily assessed by PCR than single nucleotide variants, as they create completely new sequences that are not present in normal tissue. Anyway, novel NGS-based techniques have been developed that offer higher sensitivity and throughput for ctDNA variant detection [22,32,33,107,108].

##### ALK-Positive Patients in Large Cohorts

Several large studies evaluating the diagnostic and prognostic use of ctDNA in NSCLC, that included some ALK+ patients, are available. In one of the first prospective studies using a clinical NGS panel for plasma and tissue samples from NSCLC, 102 patients were analyzed for the detection of therapeutically targetable and resistant mutations. Genetic variants (point mutations, indels and fusions) were detected in 86/102 plasma samples, including two EML4/ALK-positive patients, one of whom had undergone undetected by tissue analysis and was then successfully treated with crizotinib [128]. Overall, plasma tests detected clinically relevant mutations in 84% samples compared to 78% in tissue samples, indicating not only the utility of ctDNA but also its potential superiority for variant detection in settings where tissue DNA is not available or has poor quality.

Employing the CAPP-seq (CAncer Personalized Profiling by deep Sequencing) pull-down approach, Newman and colleagues were able to detect, among other mutations, the *EML4-ALK* fusion in a cohort of advanced NSCLC patients [30]. To assess the clinical applicability of ctDNA testing before therapy assignment, Schwaederlé et al. analyzed plasma ctDNA in 88 consecutive NSCLC patients and found that *ALK* ranked among the most frequently mutated genes (6.8% of patients), with a high concordance rate between ctDNA and tissue testing (Table 1). An appreciable therapeutic efficacy was observed in patients who received matched therapy according to the detected alteration in ctDNA: 72.3% of evaluable patients achieved durable stable disease or partial response [99]. The Actionable Genome Consortium developed an ultra-deep cfDNA NGS assay to detect driver oncogenes and resistance mechanisms from plasma samples in NSCLC patients [106]. Eight ALK+ patients were included in the study, five of whom could be detected by plasma tests (62% sensitivity and 100% specificity).

In another study aimed to establish the role of plasma genotyping in conjunction with tumor genotyping, 323 metastatic NSCLC patients were assessed for actionable targets and to guide clinical decisions. Within this large cohort, 18 patients were found to carry *ALK* mutations or fusions, including 6 patients with drug-resistant *ALK* mutations (Table 2) and one patient who was directed to alectinib therapy based on plasma analysis and achieved a partial response [100]. Similarly, a prospective study on 282 previously untreated NSCLC patients showed non-inferior sensitivity of ctDNA analysis compared to tissue genotyping in identifying actionable targets, including *ALK* fusions (NILE study, Non-invasive versus Invasive Lung Evaluation; ClinicalTrials.gov; NCT03615443). The study showed a 48% increase in biomarker detection rate with the ctDNA test compared to tissue analysis alone, including 20% of patients for which tissue was unavailable, and turnaround times were faster [101]. In this trial, concordance between tissue and plasma genotyping was 99% in 8 ALK+ and 207 tissue ALK− patients assessed for ALK fusions.

##### ALK-Focused Diagnostic Studies

Several groups have evaluated the use of ctDNA to specifically diagnose ALK+ NSCLC. Using a capture-based NGS method, Cui and colleagues assessed the use of ctDNA to detect *ALK* fusions in NSCLC patients. Although the sample size of the study was relatively small, the group reported 71.8% consistency in the detection of *ALK* rearrangement in ctDNA (Table 1). The two noteworthy findings of the study were the identification of two rare *ALK* rearrangements and a zero false-positive rate (100% specificity) of *ALK* detection in ctDNA [102]. In another study, *ALK* rearrangements were identified in the ctDNA of 19 out of 24 ALK+ NSCLC patients [89]; longitudinal follow-up showed that ctDNA detection correlated with disease progression and the authors could identify *ALK* mutations in plasma post-crizotinib. A slightly lower sensitivity (67%) was reported for the detection of *ALK*/*ROS1* fusions using amplicon-based sequencing in ctDNA of treatment-naïve NSCLC patients at the time of diagnosis [129]. The sensitivity of *ALK* fusion detection in ctDNA improves in patients at disease progression, probably due to the increase in DNA shedding in plasma of patients with advanced, aggressive disease [103,129]. In addition, novel *ALK* fusions have been identified in ctDNA: for example, in the phase I/II ensartinib study, 22 patients for whom blood and tissue genotyping was performed, three different fusion partners and five unique *EML4-ALK* fusion variants were identified [104]. Moreover, progression of disease due to the acquisition of new ALK mutations was detected prior to radiographic progression, which can save precious time to switch treatments [104].

In 53 patients from the phase III ALEX study, who had matched tissue and plasma available, the same *EML4-ALK* fusion was identified in 79% of the matched samples [105]. More recently Gadgeel and colleagues analyzed the efficacy of alectinib in a cohort of ALK+ patients enrolled by blood-based NGS (BFAST trial NCT03178552). With the limitations of cross-trial comparison, alectinib performed as expected in this blood-first screening trial, showing an ORR of 87%. Secondary biomarker analyses indicated comparable results of ctDNA and tissue-based determinations [130]. A recent case report illustrates the potential real-world use of ctDNA in this setting: following disease progression on chemotherapy, *EML4-ALK* fusion was detected in plasma ctDNA, the patient switched to alectinib and achieved a durable complete response [131]. In another case with an unusual *KLC1-ALK* fusion, plasma analysis was used to identify the fusion partner and to monitor therapy response over time. The results of ctDNA sequencing during treatment reflected the state of remission and could predict the subsequent clinical course. At the time of progression, *ALK* mutations were identified in ctDNA that potentially caused treatment failure [118]. Thus, ctDNA can be used to monitor NSCLC treatment response as a surrogate MRD marker, since tumors responding to the treatment shed less DNA in the blood [132,133]. Prospective pre-TKI plasma collection allows comparison of relapse and baseline samples, for precise therapeutic monitoring and tracking of resistance [104]. Preliminary data from the phase II ensartinib clinical trial showed that higher ctDNA amount correlated with poor PFS [134]. Longitudinal monitoring of ctDNA using ddPCR revealed worse PFS in a group of patients with detectable ctDNA in the pre-treatment samples [135].

Adding further evidence to the clinical utility of this approach, in a retrospective analysis on NSCLC plasma samples using the Guardant360 NGS gene panel, 11/42 (26%) patients whose tissue biopsy was inadequate for analysis were found positive for ALK rearrangement in cfDNA. Additionally, five patients resulted to be ALK-positive in ctDNA while tissue tested negative; of these, three received TKI based on ctDNA results and responded to therapy [136]. Supplee et al. compared two hybrid-capture NGS-based assays for the detection of *ALK*, *ROS1* or *RET* fusions in a small subset of NSCLC patients [137]. The authors reported higher sensitivity of the ctDx-Lung test compared to Guardant360 and suggested that it could be due to the use of shorter capture probes and extension primers. These results show that further technical and bioinformatics improvements will increase the clinical utility of ctDNA-based diagnostic methods in the future.

Finally, although NGS-based methods are more sensitive than conventional methods, their use is limited due to the higher cost and need for specialized equipment. As an alternative, Kunimasa et al. recently developed a targeted sequencing system using an adapter and a set of primers spanning the entire region of *ALK* intron 19 enabling PCR amplification of regions involving the breakpoint [26]. The authors validated their method using cfDNA from 20 ALK+ NSCLC and 10 healthy volunteers with 50% sensitivity and 100% specificity.

##### Analysis of Drug Resistance

While ctDNA can be used for diagnostic purposes, perhaps the biggest impact has been on identifying and monitoring resistance mechanisms in ALK+ NSCLC patients who failed targeted therapies. Using the diagnostic or pre-treatment tissue biopsy as a reference, the acquisition of new mutations in the ctDNA can be useful in guiding treatment decisions for advanced metastatic NSCLC patients (Table 2). Substantial evidence using ctDNA for the molecular profiling of ALK mutations currently exists in the literature and is continuously increasing [103,104,105,107,108,129,138,139,140,141]. Several studies have also looked at tracking the evolution of ALK kinase domain mutations as it is the most common resistance mechanism against ALK TKIs and there is a consensus on the comparability of ctDNA and tissue genotyping results [116,130,131]. For example, Dagogo-Jack and colleagues analyzed plasma and tissue specimens from 70 ALK+ patients relapsed on second- and third-generation ALK inhibitors, using the Guardant360 protocol. ALK mutations were identified in 67% and 63% of samples, respectively, but plasma analysis was more likely to provide multiple mutants, thus confirming the concept of higher clonal diversity represented in liquid versus solid biopsy [107]. The same authors ran a more comprehensive longitudinal genotyping of plasma samples from another cohort of 22 ALK+ NSCLC patients with acquired resistance to ALK TKIs. They could describe the evolution of resistance during therapy, tracking the appearance and disappearance of every ALK mutant through sequential TKI treatments [103]. As demonstrated by Shaw and colleagues, in patients exposed to lorlatinib after the failure of first/second-generation TKI, the objective response rate was higher in patients with ALK mutations in comparison to patients with no mutation (62% vs. 32%) as detected by blood-based NGS analysis [108]. At our center, a patient progressing on brigatinib was also refractory to lorlatinib and was retrospectively found to carry a compound L1196M/G1202R ALK mutation [119]. Recently, in a case where biopsy of the progressing lesion was not feasible, liquid biopsy identified a G1202R mutant clone which, following local radiotherapy, disappeared from the ctDNA [121]. Similarly, analysis of serial liquid biopsies in a patient with EML4-ALK+ NSCLC revealed two ALK mutations, G1269A and G1202R, arising during progression. Plasma levels of the mutations correlated with tumor response, demonstrating that the molecular profile of the tumor obtained from liquid biopsy allows for efficient dynamic monitoring of patients [120].

#### 3.2.3. Circulating RNA

Circulating cell-free RNA (cfRNA) comprises various species, both coding and non-coding, which are found mostly within exosomes and other extracellular vesicles, as naked RNA is highly susceptible to degradation [142]. Nevertheless, cfRNA has been used as a source of material for the detection of *ALK* fusions. Park et al. used a RT-PCR based method that was initially used for tissue genotyping: in a cohort of 61 patients (33 ALK+ and 28 ALK−), the authors reported 79% accuracy for the detection of *ALK* using cfRNA by RT-PCR [109]. One of the limitations of the study was the use of a commercial kit that can only detect known *ALK* fusions, which is not useful in cases where the rearrangement type is unknown. Moreover, to detect different variants of the *EML4-ALK* fusion, specific primers need to be designed based on the genomic fusion breakpoint location. Using the same approach, Nilsson and colleagues obtained a rather low sensitivity (21%) when probing cfRNA for fusion detection [110]. Both groups found better results using platelet-derived RNA (see below).

Among other RNA species, miRNAs have gained attention as cancer biomarkers implicating their role in pathophysiology, diagnosis and prognosis of various tumor types. In NSCLC, plasma miRNA signatures have shown prognostic value in a high-risk population [143,144,145]. Such data are more limited in the ALK+ setting and large prospective studies are warranted to establish their use as liquid biopsy biomarkers. To screen diagnostic and prognostic miRNAs in ALK+ NSCLC patients, Li et al. conducted a microarray analysis of plasma samples from a small subset of NSCLC patients (3 ALK+ and 3 ALK−) and healthy subjects [146]. The group identified 21 miRNAs that were differentially expressed in ALK+ patients. Upon further validation, 3 miRNAs (miR-28-5p, miR-362-5p, and miR-660-5p) showed the most significant difference in expression between ALK+ and ALK− patients. The 3-miRNA combination panel had 63% sensitivity, 97% specificity and an Area Under the Curve (AUC) value of 0.876 in discriminating ALK+ from ALK− patients. Changes in the level of miR-660-5p expression in plasma showed a correlation with response to crizotinib treatment. High expression of miR-362-5p was a predictor of longer PFS.

Circular RNAs (circRNAs) are a novel class of non-coding, single-stranded, covalently closed-loop RNAs that are formed predominantly as a result of the back-spliced joining of the 5′- and 3′-end of the pre-mRNA [147]. CircRNAs have gained attention due to their implication in various pathological processes including cancer. Due to their circular nature, they are resistant to exonucleases and show higher stability in plasma compared to other circulating RNAs. However, they can also be found within exosomes, which offer further protection [148]. Guarnerio and colleagues reported that oncogenic chromosomal translocations lead to the generation of fusion-circRNAs (F-circRNAs): one such F-circRNA, termed f-circEA1, is generated by the *EML4-ALK* fusion gene and was shown to promote tumor development [149]. A novel F-circEA was later detected in the plasma of 5 patients with *EML4-ALK* rearrangement, variant 3b [150]: therefore, F-circEA is a potential diagnostic liquid biopsy biomarker in EML4-ALK+ NSCLC setting. Subsequently, another F-circRNA called F-circEA-2a was identified to promote cell migration and invasion in lung cancer cells. Curiously, however, F-circEA-2a was present in the tumor but not in the plasma of NSCLC patients (*n* = 3) with the *EML4-ALK* fusion gene [151].

#### 3.2.4. Platelets

As noted above, platelets act as cellular sponges collecting tumor-derived macromolecules and can be useful in the prediction and monitoring of treatment response. *EML4-ALK* rearrangement was detected in platelets from 67 NSCLC patients with a 65% sensitivity and 100% specificity [110]. In the same study, PFS was 3.7 months for patients with EML4-ALK+ platelets and 16 months for those with EML4-ALK-negative platelets. The authors also reported higher sensitivity of detection from platelet versus plasma cfRNA. Using platelet-derived RNA, Calvo et al. verified the presence of *ALK* rearrangement during treatment with crizotinib in a NSCLC patient and quantified the fusion transcript through RT-PCR. Detection of platelet *EML4-ALK* allowed monitoring of therapeutic response and disease progression by sequential blood collection from this patient [152].

Comparing liquid biopsy from plasma and platelets versus FISH/RT-PCR tests performed on FFPE tumor tissues for the detection of *ALK* rearrangement and prediction of treatment outcome, it was found that liquid biopsy had greater sensitivity (78.8% vs. 54.5%), specificity (89.3% vs. 78.6%) and accuracy (83.6% vs. 75.5%). Additionally, platelets exhibited slightly higher sensitivity in detection and superior predictability of treatment results compared to plasma [109]. These results indicate that platelets may better reflect the molecular state of tumor tissue than plasma.

Taken together, the above-mentioned studies potentiate the role of TEPs as liquid biopsy biomarkers in ALK+ NSCLC; however, in order to implement this approach in the clinic, a few limitations still need to be overcome, such as standardization of TEPs detection and accessibility of this technique in hospitals.

#### 3.2.5. Exosomes

Increasing evidence is being reported in support of the role exosomes play in tumor biology and particularly in NSCLC. They can promote tumor growth, angiogenesis, invasion and metastasis, leading to progression of NSCLC [153,154,155,156,157]. Exosomes can also render tumor cells resistant to targeted therapies by transferring tissue factors, drug-resistant molecules or multi-drug resistance proteins [158,159].

Brinkmann and colleagues reported using a proprietary method to isolate exosomal RNA (exoRNA) from less than 2 mL of plasma from NSCLC patients. The extracted exoRNA was screened for the presence of *EML4-ALK* fusion transcripts using RT-qPCR [160]. The assay was launched in 2016 in the US as a commercial diagnostic test kit (ExoDx^®^ Lung(ALK)) to detect EML4-ALK fusion variants in blood samples. The same group has also reported analysis of ALK resistance mutations from exoRNA and cfDNA in 35 longitudinal samples of 29 patients [122]. Another group led by Christian Rolfo reported the identification of *EML4-ALK* inversion in exosomes (ExoALK) from 1 mL plasma samples using next-generation sequencing: 19 NSCLC patients, out of which 16 were confirmed ALK+ in tissue, were evaluated. The authors reported 64% concordance between tissue and exosomal analysis. All three patients that were negative in tissue were also negative in exosomal analysis indicating high specificity (100%) of exosomal RNA for the detection of *ALK* rearrangement [111]. Encouraged by these results, a clinical trial is currently underway in NSCLC patients with the aim to evaluate the performance of exosomal-based *EML4-ALK* fusion detection in comparison to IHC-based detection of the rearrangement in tissue. The study will also monitor changes in *EML4-ALK* fusion in exosomes in pre- and post-treatment samples as well as the prognostic potential of exosome-based *EML4-ALK* detection (ClinicalTrial Identifier: NCT04499794).

Collectively, these studies indicate exosomes as an exciting source of information for liquid biopsy in ALK-driven NSCLC. Further improvements in exosome isolation techniques and larger controlled studies exploring the use of exosome as biomarkers will help substantiate their use as liquid biopsy biomarkers.

### 3.3. Neuroblastoma and Other ALK+ Tumors

Neuroblastoma is the most common extracranial solid malignancy in children. It is characterized by high genetic and phenotypic heterogeneity, ranging from spontaneous regression to highly aggressive disease. Patients with low-risk disease are monitored by observation, while patients with high-risk tumors need high-intensity chemotherapy, with low long-term survival rates. Monitoring of neuroblastoma is normally performed by tumor biopsy, imaging, and bone marrow aspirates. For high-risk patients, there are no established blood biomarkers to monitor the response to therapy. As neuroblastoma often overexpresses (and is driven by) the *MYCN* oncogene, detection of *MYCN* amplification through plasma DNA sequencing has been investigated by several labs [161,162,163,164,165]. The data collectively suggested that *MYCN* liquid biopsy could allow patients stratification and monitoring, as well as outcome prediction. A fraction (up to 10%) of sporadic neuroblastomas and virtually all familial cases are characterized by *ALK* activating point mutations or gene amplification [166,167]. Indeed, the concomitant expression of MYCN and ALK^F1174L^ causes neuroblastoma *in vivo* from neural crest cells [168]. Therefore, ddPCR analysis was developed for the simultaneous detection of *MYCN* and *ALK* gene copy numbers from cfDNA [169]. The data suggested that ddPCR can reliably detect amplification in gDNA from a 1:10 mixture of neuroblastoma cells in a background of non-amplified cells. Furthermore, the authors could correctly identify *MYCN* and *ALK* amplification or diploid status in plasma samples from mice with established neuroblastoma xenografts and from patients at diagnosis, in accordance with FISH results on the primary tumor. In few cases, a higher copy number was detected by ctDNA compared to primary biopsy, which may reflect the presence of more aggressive metastatic clones that are not detected by tissue biopsy, or heterogeneous primary tumor tissue that is not appreciated by single regional sampling. In a further technical development, the same group described a quadruplexed ddPCR protocol to quantify *MYCN* and *ALK* copy number together with two reference genes, and simultaneously estimate *ALK* mutant allele frequency in the circulating DNA [170]. Similarly, *MYCN* and *ALK* copy number alterations (CNAs) were monitored by cfDNA analysis by Kobayashi and co-workers in *MYCN/ALK* co-amplified cases using a simple qPCR approach; the authors suggested that *MYCN/ALK* CNAs can be employed as molecular biomarkers in this population [171]. Combaret et al. developed a ddPCR protocol to detect *ALK* hotspot variants (Table 2) in ctDNA from neuroblastoma patients, using mutation-specific probes [123]. The method displayed high sensitivity and specificity, with perfect concordance between plasma and tumor samples. Droplet digital PCR outperformed Sanger sequencing and compared well with deep sequencing in primary tumor analysis, but the most important result was the fact that small amounts of plasma (200 µL) could be used in the ddPCR screening, which makes this technique very convenient in this setting, where doctors deal with very young patients. In an elegant longitudinal study, Chicard et al. ran whole-exome sequencing (WES) from cfDNA of 19 neuroblastoma patients at different time points during therapy [124]. By comparing cfDNA at diagnosis with post-relapse samples, the authors could identify relapse-specific variants. Genes recurrently found mutated at diagnosis in cfDNA included *ALK*. In one case, the *ALK* variant disappeared at the time of complete remission; in another patient, the same *ALK* mutation was conserved between diagnosis and relapse. In general, on average, 22 alterations per patient were unique to the relapse sample and may explain progression, including *KRAS* mutations and *CDK4/6* amplifications, although deeper coverage revealed that in some cases these variants were present as minor subclones also in the initial sample. Allelic frequencies were used to infer clonal evolution in two cases. These data show that the analysis of circulating DNA offers a great opportunity to describe evolutionary dynamics in tumors and to take action for better therapy outcomes. An alternative to WES is represented by targeted panels, especially when searching for actionable mutations: Cimmino and colleagues tested the value of a gene panel for the detection of variants associated with neuroblastoma in ctDNA samples, which could be specifically targeted by approved drugs. Mutations were identified in the majority of patients (9 of 11 [82%]), including pathogenic variants of *ALK*, *FGFR1* and *NOTCH1* [125].

In a different disease setting, ctDNA analysis of a patient with prostate carcinoma identified an ALK F1174C mutation, confirmed in the primary tissue. This allowed treatment with alectinib, resulting in stable disease and reduction of mutated *ALK* allele fraction in the ctDNA [126]. A recent investigation of the genomic landscape of metastatic papillary thyroid carcinoma showed that fusion-positive patients (including an EML4/ALK case) were significantly more likely to develop distant metastasis and that plasma ctDNA detection rate was significantly associated with metastatic disease [172].

In a large cohort of colorectal cancer patients, ctDNA analysis allowed the identification of actionable gene fusions, including 10 ALK fusion-positive patients; 7/10 samples also carried additional mutations in *EGFR*, *KRAS* and *NRAS* genes and were associated with resistance to anti-EGFR therapy [173]. Interestingly, this anti-EGFR signature was associated with lower frequency of the co-occurring alterations, which points to a subclonal architecture of the advanced disease, which may have been missed by primary tissue analyses. The group led by Dr. Bardelli reported monitoring of a metastatic colorectal cancer patient with a *CAD-ALK* fusion using cfDNA from urine and plasma, during treatment with entrectinib [127]. Digital PCR levels of fusion detection in liquid samples anticipated clinical response and allowed the identification of resistance mutations.

Finally, a recent case report described CTCs detection in an ALK+ IMT patient [174]. CTCs stained positive for ALK protein and were only seen before TKI therapy: nine months under entrectinib, no CTCs were found in the blood, in parallel with complete radiological remission.

## 4. Conclusions

It is now clear from several studies that genetic information of tumors can be obtained in a non-invasive manner from blood. This information has diagnostic, prognostic and predictive utility, and can also provide clues on the subclonal heterogeneity of tumors, by collecting genetic data from both primary and metastatic sites. Different methodologies have been put in place during the last few years, each of which has advantages and pitfalls. The sensitivity of these approaches can vary according to the method and the amount of DNA shed by a tumor into the bloodstream. From traditional quantitative PCR, technology moved to droplet digital PCR, multiplexed amplicon deep sequencing and hybridization-based NGS methods, and the field is rapidly advancing.

Several sources of liquid biopsy can be used: tumor DNA and RNA can be obtained from plasma, urine, purified exosomes, or platelets. As an alternative, CTCs can be analyzed. While ctDNA analysis is cheaper and more straightforward, making it a good candidate for clinical practice, CTCs provide single-cell information, offering the possibility to investigate tumor heterogeneity and therapeutic resistance at the subclonal level. Moreover, CTCs allow researchers to investigate the biology of the metastatic process. However, a global consensus on CTC isolation procedures is still lacking and sensitivity must be increased, as shown by the recent AIR trial, before CTC detection becomes a routine clinical tool.

ALK fusion-positive cancers carry a specific aberration that represents a perfect marker for disease detection and monitoring: in this regard, it may be easier for *ALK*-rearranged tumors to reach routine use of these novel tools, for better management of patients, compared to other, non-translocated cancers. Most of the studies up to now have reported high specificity for ctDNA-based *ALK* rearrangement detection; however, sensitivity still needs to be improved. Reduced sensitivity can be attributed to multiple factors, such as low disease burden or low DNA shedding in the bloodstream.

The prognostic value of *ALK* fusion detection has been established in ALCL patients, where it is used as an MRD biomarker. However, the largest amount of data is found in the NSCLC setting, where the utility of liquid biopsy was demonstrated not only for diagnostic purposes, but also for investigating drug resistance mechanisms. Recent advances in this technology led to the FDA approval of the Guardant360^®^ CDx test, analyzing ctDNA variants in 73 genes plus fusions and copy-number alterations in selected guideline-recommended genes including *ALK*. Liquid biopsy in ALK+ tumors gives the physician the chance (i) to follow the course of therapy response by simple PCR detection of the fusion, identifying molecular relapses several weeks before clinical evidence; and (ii) to identify TKI-resistant ALK mutants (or off-target mutations) in order to switch therapy in advance. In ALK+ ALCL, where therapy shows high cure rates, liquid biopsy can identify high-risk patients with minimal residual disease that is radiographically invisible.

Sparse information is available for additional, rarer ALK+ tumors like neuroblastoma, colon, prostate, thyroid, IMT. No liquid biopsy data on other ALK+ cancer types, such as renal carcinoma, have been found in our literature search.

## Figures and Tables

**Table 1:** Results obtained by liquid biopsy in a diagnostic setting, in ALK+ NSCLC patients. Tissue genotyping is used as a reference.

Study	Ref.	Tumor	Material	*N*. Patients	Method	Sensitivity	Specificity	Accuracy
Wang et al.	[89]	NSCLC	ctDNA	24	capture	79%	100%	92%
Ilie et al.	[94]	NSCLC	CTC	87	FISH, ICC	100%	--	100%
Pailler et al.	[95]	NSCLC	CTC	32	FISH	100%	100%	100%
Tan et al.	[96]	NSCLC	CTC	26	FISH	--	--	90%
Ilie et al.	[98]	NSCLC	CTC	203	ICC	94%	89%	84%
					FISH	36%	57%	21%
Schwaederlé et al.	[99]	NSCLC	ctDNA	88	capture, PCR	64%	64%	81%
Aggarwal et al.	[100]	NSCLC	ctDNA	18 *	capture, PCR	72%	79%	75%
Leighl et al.	[101]	NSCLC	ctDNA	8 ^†^	capture	75%	100%	99%
Cui et al.	[102]	NSCLC	ctDNA	24	capture	54%	100%	72%
Dagogo-Jack et al.	[103]	NSCLC	ctDNA	22	capture	86%	--	100%
Horn et al.	[104]	NSCLC	ctDNA	76	capture	91%	--	91%
Camidge et al.	[105]	NSCLC	ctDNA	53	capture	--	--	79%
Li et al.	[106]	NSCLC	ctDNA	8	capture, PCR	62%	100%	62%
Dagogo-Jack et al.	[107]	NSCLC	ctDNA	15	capture	90%	48%	68%
Shaw et al.	[108]	NSCLC	ctDNA	198	capture	61%	82%	73%
Park et al.	[109]	NSCLC	cfRNA	66	PCR	64%	96%	79%
			platelets	26	PCR	70%	93%	80%
			cfRNA+platelets	61	PCR	79%	89%	84%
Nilsson et al.	[110]	NSCLC	cfRNA	32	PCR	21%	100%	66%
			platelets	67	PCR	65%	100%	86%
Reclusa et al.	[111]	NSCLC	exosomes	17	PCR	64%	100%	71%

* from a cohort of 128 NSCLC patients. ^†^ from a cohort of 215 NSCLC patients.

**Table 2:** ALK mutational data obtained by liquid biopsy in various ALK+tumors.

Study	Ref.	Tumor	Material	Method	Variant(s)	Therapy
Gambacorti-Passerini et al.	[54]	ALCL	RNA ^†^	PCR	I1171N, M1328I	Crizotinib
Wang et al.	[89]	NSCLC	ctDNA	capture	L1152R, I1171T, L1196M	Crizotinib
Zhang et al.	[116]	NSCLC	CTC	PCR	L1196M	Crizotinib
Pailler et al.	[117]	NSCLC	CTC	PCR	G1202R+F1174C, G1202R+T1151M	Lorlatinib
Aggarwal et al.	[100]	NSCLC	ctDNA	capture, PCR	I1171S/T, F1174L, L1196Q, G1202R, G1123S	n.r.
Dagogo-Jack et al.	[103]	NSCLC	ctDNA	capture	I1171N, F1174C/L, L1196M, G1202R, D1203N, E1210K, I1171N+G1202R	Ceritinib, Alectinib, Brigatinib
Horn et al.	[104]	NSCLC	ctDNA	capture	G1269A, S1206F, G1202R; others at low freq.	Crizotinib or Crizotinib + 2nd gen. TKI
Dietz et al.	[118]	NSCLC	ctDNA	capture	F1174C/L, G1269A	Crizotinib
Dagogo-Jack et al.	[107]	NSCLC	ctDNA	capture	G1202R, L1196M, I1171N/T/S, V1180L, G1269A, D1203N, G1202R+L1196M, D1203N+I1171N; others at low freq.	Multiple sequential TKIs
Shaw et al.	[108]	NSCLC	ctDNA	capture	G1269A, G1202R, F1174X *, L1196M, I1171X *; others at low freq.	Various TKIs
Sharma et al.	[119]	NSCLC	ctDNA	PCR	L1196M+G1202R	Brigatinib
Sánchez-Herrero et al.	[120]	NSCLC	ctDNA	PCR	G1269A, G1202R	Crizotinib and Ceritinib
König et al.	[121]	NSCLC	ctDNA	n.r.	G1202R	Ceritinib
Brinkmann et al.	[122]	NSCLC	exosomes	PCR	L1196M, G1202R	n.r.
Combaret et al.	[123]	NB	ctDNA	PCR	F1174L, R1275Q	Pre-treatment
Chicard et al.	[124]	NB	ctDNA	capture	H1030P, F1174L, L1196M	Pre-treatment, chemo
Cimmino et al.	[125]	NB	ctDNA	capture	F1174L	Pre-treatment
Carneiro et al.	[126]	PCa	ctDNA	capture	F1174C	Pre-treatment
Siravegna et al.	[127]	CRC	ctDNA	PCR	F1174L/C, G1128A, F1245V	Entrectinib

^†^ total RNA obtained from peripheral blood mononuclear cells. * X indicates several possible substitutions; NB, neuroblastoma; PCa, prostate carcinoma; CRC, colorectal cancer; n.r., not reported.

## References

[1] Mologni L., Friboulet L. (2021). Resistance mechanisms to ALK TKIs in tumors other than lung cancer. Therapeutic Strategies to Overcome ALK Resistance in Cancer.

[2] Sharma G.G., Mota I., Mologni L., Patrucco E., Gambacorti-Passerini C., Chiarle R. (2018). Tumor resistance against ALK targeted therapy-Where it comes from and where it goes. Cancers.

[3] Zugazagoitia J., Ramos I., Trigo J.M., Palka M., Gómez-Rueda A., Jantus-Lewintre E., Camps C., Isla D., Iranzo P., Ponce-Aix S. (2019). Clinical utility of plasma-based digital next-generation sequencing in patients with advance-stage lung adenocarcinomas with insufficient tumor samples for tissue genotyping. Ann. Oncol..

[4] Rolfo C., Mack P.C., Scagliotti G.V., Baas P., Barlesi F., Bivona T.G., Herbst R.S., Mok T.S., Peled N., Pirker R. (2018). Liquid Biopsy for Advanced Non-Small Cell Lung Cancer (NSCLC): A Statement Paper from the IASLC. J. Thorac. Oncol..

[5] Chaudhuri A.A., Chabon J.J., Lovejoy A.F., Newman A.M., Stehr H., Azad T.D., Khodadoust M.S., Esfahani M.S., Liu C.L., Zhou L. (2017). Early detection of molecular residual disease in localized lung cancer by circulating tumor DNA profiling. Cancer Discov..

[6] Gupta G.P., Massagué J. (2006). Cancer metastasis: Building a framework. Cell.

[7] Elazezy M., Joosse S.A. (2018). Techniques of using circulating tumor DNA as a liquid biopsy component in cancer management. Comput. Struct. Biotechnol. J..

[8] Meng S., Tripathy D., Frenkel E.P., Shete S., Naftalis E.Z., Huth J.F., Beitsch P.D., Leitch M., Hoover S., Euhus D. (2004). Circulating tumor cells in patients with breast cancer dormancy. Clin. Cancer Res..

[9] Tamminga M., Groen H.H.J.M., Hiltermann T.J.N. (2016). Investigating CTCs in NSCLC-a reaction to the study of Jia-Wei Wan: A preliminary study on the relationship between circulating tumor cells count and clinical features in patients with non-small cell lung cancer. J. Thorac. Dis..

[10] Cristofanilli M., Budd G.T., Ellis M.J., Stopeck A., Matera J., Miller M.C., Reuben J.M., Doyle G.V., Allard W.J., Terstappen L.W.M.M. (2004). Circulating tumor cells, disease progression, and survival in metastatic breast cancer. N. Engl. J. Med..

[11] Allard W.J., Matera J., Miller M.C., Repollet M., Connelly M.C., Rao C., Tibbe A.G.J., Uhr J.W., Terstappen L.W.M.M. (2004). Tumor cells circulate in the peripheral blood of all major carcinomas but not in healthy subjects or patients with nonmalignant diseases. Clin. Cancer Res..

[12] de Bono J.S., Scher H.I., Montgomery R.B., Parker C., Miller M.C., Tissing H., Doyle G.V., Terstappen L.W.W.M., Pienta K.J., Raghavan D. (2008). Circulating tumor cells predict survival benefit from treatment in metastatic castration-resistant prostate cancer. Clin. Cancer Res..

[13] Hanssen A., Wagner J., Gorges T.M., Taenzer A., Uzunoglu F.G., Driemel C., Stoecklein N.H., Knoefel W.T., Angenendt S., Hauch S. (2016). Characterization of different CTC subpopulations in non-small cell lung cancer. Sci. Rep..

[14] Aya-Bonilla C.A., Marsavela G., Freeman J.B., Lomma C., Frank M.H., Khattak M.A., Meniawy T.M., Millward M., Warkiani M.E., Gray E.S. (2017). Isolation and detection of circulating tumour cells from metastatic melanoma patients using a slanted spiral microfluidic device. Oncotarget.

[15] Kulasinghe A., Kapeleris J., Cooper C., Warkiani M.E., O’Byrne K., Punyadeera C. (2019). Phenotypic Characterization of Circulating Lung Cancer Cells for Clinically Actionable Targets. Cancers.

[16] Mendel P., Mandel L. (1948). On the comparative behavior, during the prolonged protein fast, of the two nuclear acids of animal tissues and on its significance. C. R. Hebd. Seances Acad. Sci..

[17] Shaw J.A., Stebbing J. (2014). Circulating free DNA in the management of breast cancer. Ann. Transl. Med..

[18] Diaz L.A.J., Bardelli A. (2014). Liquid biopsies: Genotyping circulating tumor DNA. J. Clin. Oncol..

[19] Yong E. (2014). Cancer biomarkers: Written in blood. Nature.

[20] Jahr S., Hentze H., Englisch S., Hardt D., Fackelmayer F.O., Hesch R.D., Knippers R. (2001). DNA fragments in the blood plasma of cancer patients: Quantitations and evidence for their origin from apoptotic and necrotic cells. Cancer Res..

[21] Diehl F., Schmidt K., Choti M.A., Romans K., Goodman S., Li M., Thornton K., Agrawal N., Sokoll L., Szabo S.A. (2008). Circulating mutant DNA to assess tumor dynamics. Nat. Med..

[22] Guibert N., Hu Y., Feeney N., Kuang Y., Plagnol V., Jones G., Howarth K., Beeler J.F., Paweletz C.P., Oxnard G.R. (2018). Amplicon-based next-generation sequencing of plasma cell-free DNA for detection of driver and resistance mutations in advanced non-small cell lung cancer. Ann. Oncol..

[23] Paweletz C.P., Sacher A.G., Raymond C.K., Alden R.S., O’Connell A., Mach S.L., Kuang Y., Gandhi L., Kirschmeier P., English J.M. (2016). Bias-Corrected Targeted Next-Generation Sequencing for Rapid, Multiplexed Detection of Actionable Alterations in Cell-Free DNA from Advanced Lung Cancer Patients. Clin. Cancer Res..

[24] Kukita Y., Matoba R., Uchida J., Hamakawa T., Doki Y., Imamura F., Kato K. (2015). High-fidelity target sequencing of individual molecules identified using barcode sequences: De novo detection and absolute quantitation of mutations in plasma cell-free DNA from cancer patients. DNA Res. Int. J. Rapid Publ. Rep. Genes Genomes.

[25] Shi Y.F., Liu C.L., Zhou C.J., Gong L.P., Dong L.N., Li M., Huang X., Gao Z.F. (2008). Anaplastic lymphoma kinase gene abnormality and the expression of its fusion protein in primary systemic anaplastic large cell lymphoma. Beijing Da Xue Xue Bao.

[26] Kunimasa K., Kato K., Imamura F., Kukita Y. (2019). Quantitative detection of ALK fusion breakpoints in plasma cell-free DNA from patients with non-small cell lung cancer using PCR-based target sequencing with a tiling primer set and two-step mapping/alignment. PLoS ONE.

[27] Maheswaran S., Sequist L.V., Nagrath S., Ulkus L., Brannigan B., Collura C.V., Inserra E., Diederichs S., Iafrate A.J., Bell D.W. (2008). Detection of mutations in EGFR in circulating lung-cancer cells. N. Engl. J. Med..

[28] Diehl F., Li M., He Y., Kinzler K.W., Vogelstein B., Dressman D. (2006). BEAMing: Single-molecule PCR on microparticles in water-in-oil emulsions. Nat. Methods.

[29] Kuang Y., Rogers A., Yeap B.Y., Wang L., Makrigiorgos M., Vetrand K., Thiede S., Distel R.J., Jänne P.A. (2009). Noninvasive detection of EGFR T790M in gefitinib or erlotinib resistant non-small cell lung cancer. Clin. Cancer Res..

[30] Newman A.M., Bratman S.V., To J., Wynne J.F., Eclov N.C.W., Modlin L.A., Liu C.L., Neal J.W., Wakelee H.A., Merritt R.E. (2014). An ultrasensitive method for quantitating circulating tumor DNA with broad patient coverage. Nat. Med..

[31] Couraud S., Vaca-Paniagua F., Villar S., Oliver J., Schuster T., Blanché H., Girard N., Trédaniel J., Guilleminault L., Gervais R. (2014). Noninvasive diagnosis of actionable mutations by deep sequencing of circulating free DNA in lung cancer from never-smokers: A proof-of-concept study from BioCAST/IFCT-1002. Clin. Cancer Res..

[32] Oxnard G.R., Paweletz C.P., Kuang Y., Mach S.L., O’Connell A., Messineo M.M., Luke J.J., Butaney M., Kirschmeier P., Jackman D.M. (2014). Noninvasive detection of response and resistance in EGFR-mutant lung cancer using quantitative next-generation genotyping of cell-free plasma DNA. Clin. Cancer Res..

[33] Newman A.M., Lovejoy A.F., Klass D.M., Kurtz D.M., Chabon J.J., Scherer F., Stehr H., Liu C.L., Bratman S.V., Say C. (2016). Integrated digital error suppression for improved detection of circulating tumor DNA. Nat. Biotechnol..

[34] Mitchell P.S., Parkin R.K., Kroh E.M., Fritz B.R., Wyman S.K., Pogosova-Agadjanyan E.L., Peterson A., Noteboom J., O’Briant K.C., Allen A. (2008). Circulating microRNAs as stable blood-based markers for cancer detection. Proc. Natl. Acad. Sci. USA.

[35] Chen X., Ba Y., Ma L., Cai X., Yin Y., Wang K., Guo J., Zhang Y., Chen J., Guo X. (2008). Characterization of microRNAs in serum: A novel class of biomarkers for diagnosis of cancer and other diseases. Cell Res..

[36] Bartel D.P. (2004). MicroRNAs: Genomics, biogenesis, mechanism, and function. Cell.

[37] Esquela-Kerscher A., Slack F.J. (2006). Oncomirs—microRNAs with a role in cancer. Nat. Rev. Cancer.

[38] Garzon R., Calin G.A., Croce C.M. (2009). MicroRNAs in Cancer. Annu. Rev. Med..

[39] Calin G.A., Croce C.M. (2006). MicroRNA signatures in human cancers. Nat. Rev. Cancer.

[40] Mumford S.L., Towler B.P., Pashler A.L., Gilleard O., Martin Y., Newbury S.F. (2018). Circulating MicroRNA Biomarkers in Melanoma: Tools and Challenges in Personalised Medicine. Biomolecules.

[41] Taverna S., Giallombardo M., Gil-Bazo I., Carreca A.P., Castiglia M., Chacártegui J., Araujo A., Alessandro R., Pauwels P., Peeters M. (2016). Exosomes isolation and characterization in serum is feasible in non-small cell lung cancer patients: Critical analysis of evidence and potential role in clinical practice. Oncotarget.

[42] Li J., Tian T., Zhou X. (2019). The role of exosomal shuttle RNA (esRNA) in lymphoma. Crit. Rev. Oncol. Hematol..

[43] Mittelbrunn M., Sánchez-Madrid F. (2012). Intercellular communication: Diverse structures for exchange of genetic information. Nat. Rev. Mol. Cell Biol..

[44] Wang C., Wang A., Wei F., Wong D.T.W., Tu M. (2017). Electric Field-Induced Disruption and Releasing Viable Content from Extracellular Vesicles. Methods Mol. Biol..

[45] Demory Beckler M., Higginbotham J.N., Franklin J.L., Ham A.-J., Halvey P.J., Imasuen I.E., Whitwell C., Li M., Liebler D.C., Coffey R.J. (2013). Proteomic analysis of exosomes from mutant KRAS colon cancer cells identifies intercellular transfer of mutant KRAS. Mol. Cell. Proteomics.

[46] Chen Y., Guo W., Fan J., Chen Y., Zhang X., Chen X., Luo P. (2017). The applications of liquid biopsy in resistance surveillance of anaplastic lymphoma kinase inhibitor. Cancer Manag. Res..

[47] Kuznetsov H.S., Marsh T., Markens B.A., Castaño Z., Greene-Colozzi A., Hay S.A., Brown V.E., Richardson A.L., Signoretti S., Battinelli E.M. (2012). Identification of luminal breast cancers that establish a tumor-supportive macroenvironment defined by proangiogenic platelets and bone marrow-derived cells. Cancer Discov..

[48] Calverley D.C., Phang T.L., Choudhury Q.G., Gao B., Oton A.B., Weyant M.J., Geraci M.W. (2010). Significant downregulation of platelet gene expression in metastatic lung cancer. Clin. Transl. Sci..

[49] Nilsson R.J.A., Balaj L., Hulleman E., van Rijn S., Pegtel D.M., Walraven M., Widmark A., Gerritsen W.R., Verheul H.M., Vandertop W.P. (2011). Blood platelets contain tumor-derived RNA biomarkers. Blood.

[50] Best M.G., Sol N., Kooi I., Tannous J., Westerman B.A., Rustenburg F., Schellen P., Verschueren H., Post E., Koster J. (2015). RNA-Seq of Tumor-Educated Platelets Enables Blood-Based Pan-Cancer, Multiclass, and Molecular Pathway Cancer Diagnostics. Cancer Cell.

[51] Ferreri A.J.M., Govi S., Pileri S.A., Savage K.J. (2012). Anaplastic large cell lymphoma, ALK-positive. Crit. Rev. Oncol. Hematol..

[52] Prokoph N., Larose H., Lim M.S., Burke G.A.A., Turner S.D. (2018). Treatment options for paediatric anaplastic large cell lymphoma (ALCL): Current standard and beyond. Cancers.

[53] Morris S.W., Kirstein M.N., Valentine M.B., Dittmer K.G., Shapiro D.N., Saltman D.L., Look A.T. (1994). Fusion of a kinase gene, ALK, to a nucleolar protein gene, NPM, in non-Hodgkin’s lymphoma. Science.

[54] Gambacorti Passerini C., Farina F., Stasia A., Redaelli S., Ceccon M., Mologni L., Messa C., Guerra L., Giudici G., Sala E. (2014). Crizotinib in advanced, chemoresistant anaplastic lymphoma kinase-positive lymphoma patients. J. Natl. Cancer Inst..

[55] Mosse Y.P., Voss S.D., Lim M.S., Rolland D., Minard C.G., Fox E., Adamson P., Wilner K., Blaney S.M., Weigel B.J. (2017). Targeting ALK With Crizotinib in Pediatric Anaplastic Large Cell Lymphoma and Inflammatory Myofibroblastic Tumor: A Children’s Oncology Group Study. J. Clin. Oncol..

[56] Fukano R., Mori T., Sekimizu M., Choi I., Kada A., Saito A.M., Asada R., Takeuchi K., Terauchi T., Tateishi U. (2020). Alectinib for relapsed or refractory anaplastic lymphoma kinase-positive anaplastic large cell lymphoma: An open-label phase II trial. Cancer Sci..

[57] Mussolin L., Pillon M., d’Amore E.S., Santoro N., Lombardi A., Fagioli F., Zanesco L., Rosolen A. (2005). Prevalence and clinical implications of bone marrow involvement in pediatric anaplastic large cell lymphoma. Leukemia.

[58] Damm-Welk C., Busch K., Burkhardt B., Schieferstein J., Viehmann S., Oschlies I., Klapper W., Zimmermann M., Harbott J., Reiter A. (2007). Prognostic significance of circulating tumor cells in bone marrow or peripheral blood as detected by qualitative and quantitative PCR in pediatric NPM-ALK-positive anaplastic large-cell lymphoma. Blood.

[59] Mussolin L., Damm-Welk C., Pillon M., Zimmermann M., Franceschetto G., Pulford K., Reiter A., Rosolen A., Woessmann W. (2013). Use of minimal disseminated disease and immunity to NPM-ALK antigen to stratify ALK-positive ALCL patients with different prognosis. Leukemia.

[60] Iijima-Yamashita Y., Mori T., Nakazawa A., Fukano R., Takimoto T., Tsurusawa M., Kobayashi R., Horibe K. (2018). Prognostic impact of minimal disseminated disease and immune response to NPM-ALK in Japanese children with ALK-positive anaplastic large cell lymphoma. Int. J. Hematol..

[61] Damm-Welk C., Mussolin L., Zimmermann M., Pillon M., Klapper W., Oschlies I., d’Amore E.S.G., Reiter A., Woessmann W., Rosolen A. (2014). Early assessment of minimal residual disease identifies patients at very high relapse risk in NPM-ALK-positive anaplastic large-cell lymphoma. Blood.

[62] Damm-Welk C., Schieferstein J., Schwalm S., Reiter A., Woessmann W. (2007). Flow cytometric detection of circulating tumour cells in nucleophosmin/anaplastic lymphoma kinase-positive anaplastic large cell lymphoma: Comparison with quantitative polymerase chain reaction. Br. J. Haematol..

[63] Damm-Welk C., Kutscher N., Zimmermann M., Attarbaschi A., Schieferstein J., Knörr F., Oschlies I., Klapper W., Woessmann W. (2020). Quantification of minimal disseminated disease by quantitative polymerase chain reaction and digital polymerase chain reaction for NPM-ALK as a prognostic factor in children with anaplastic large cell lymphoma. Haematologica.

[64] Rigaud C., Abbas R., Grand D., Minard-Colin V., Aladjidi N., Buchbinder N., Garnier N., Plat G., Couec M.-L., Duplan M. (2021). Should treatment of ALK-positive anaplastic large cell lymphoma be stratified according to minimal residual disease?. Pediatr. Blood Cancer.

[65] Lowe E.J., Reilly A.F., Lim M.S., Gross T.G., Saguilig L., Barkauskas D.A., Wu R., Alexander S., Bollard C.M. (2021). Brentuximab vedotin in combination with chemotherapy for pediatric patients with ALK+ ALCL: Results of COG trial ANHL12P1. Blood.

[66] Quelen C., Grand D., Sarot E., Brugières L., Sibon D., Pradines A., Laurent C., Brousset P., Lamant L. (2021). Minimal Residual Disease Monitoring Using a 3’ALK Universal Probe Assay in ALK-Positive Anaplastic Large-Cell Lymphoma: ddPCR, an Attractive Alternative Method to Real-Time Quantitative PCR. J. Mol. Diagn..

[67] Krumbholz M., Woessmann W., Zierk J., Seniuk D., Ceppi P., Zimmermann M., Singh V.K., Metzler M., Damm-Welk C. (2018). Characterization and diagnostic application of genomic NPM-ALK fusion sequences in anaplastic large-cell lymphoma. Oncotarget.

[68] Mussolin L., Damm-Welk C., Pillon M., Woessmann W. (2021). Minimal Disease Monitoring in Pediatric Non-Hodgkin’s Lymphoma: Current Clinical Application and Future Challenges. Cancers.

[69] Garbin A., Lovisa F., Holmes A.B., Damanti C.C., Gallingani I., Carraro E., Accordi B., Veltri G., Pizzi M., d’Amore E.S.G. (2021). miR-939 acts as tumor suppressor by modulating JUNB transcriptional activity in pediatric anaplastic large cell lymphoma. Haematologica.

[70] Merkel O., Hamacher F., Laimer D., Sifft E., Trajanoski Z., Scheideler M., Egger G., Hassler M.R., Thallinger C., Schmatz A. (2010). Identification of differential and functionally active miRNAs in both anaplastic lymphoma kinase (ALK)+ and ALK- anaplastic large-cell lymphoma. Proc. Natl. Acad. Sci. USA.

[71] Merkel O., Hamacher F., Griessl R., Grabner L., Schiefer A.-I., Prutsch N., Baer C., Egger G., Schlederer M., Krenn P.W. (2015). Oncogenic role of miR-155 in anaplastic large cell lymphoma lacking the t(2;5) translocation. J. Pathol..

[72] Hoareau-Aveilla C., Quelen C., Congras A., Caillet N., Labourdette D., Dozier C., Brousset P., Lamant L., Meggetto F. (2019). miR-497 suppresses cycle progression through an axis involving CDK6 in ALK-positive cells. Haematologica.

[73] Lovisa F., Di Battista P., Gaffo E., Damanti C.C., Garbin A., Gallingani I., Carraro E., Pillon M., Biffi A., Bortoluzzi S. (2020). RNY4 in Circulating Exosomes of Patients With Pediatric Anaplastic Large Cell Lymphoma: An Active Player?. Front. Oncol..

[74] Lovisa F., Garbin A., Crotti S., Di Battista P., Gallingani I., Damanti C.C., Tosato A., Carraro E., Pillon M., Mafakheri E. (2021). Increased Tenascin C, Osteopontin and HSP90 Levels in Plasmatic Small Extracellular Vesicles of Pediatric ALK-Positive Anaplastic Large Cell Lymphoma: New Prognostic Biomarkers?. Diagnostics.

[75] Sung H., Ferlay J., Siegel R.L., Laversanne M., Soerjomataram I., Jemal A., Bray F. (2021). Global Cancer Statistics 2020: GLOBOCAN Estimates of Incidence and Mortality Worldwide for 36 Cancers in 185 Countries. CA. Cancer J. Clin..

[76] Soda M., Choi Y.L., Enomoto M., Takada S., Yamashita Y., Ishikawa S., Fujiwara S., Watanabe H., Kurashina K., Hatanaka H. (2007). Identification of the transforming EML4-ALK fusion gene in non-small-cell lung cancer. Nature.

[77] Choi Y.L., Takeuchi K., Soda M., Inamura K., Togashi Y., Hatano S., Enomoto M., Hamada T., Haruta H., Watanabe H. (2008). Identification of novel isoforms of the EML4-ALK transforming gene in non-small cell lung cancer. Cancer Res.

[78] Mologni L. (2012). Inhibitors of the anaplastic lymphoma kinase. Expert Opin. Investig. Drugs.

[79] Just P.-A., Cazes A., Audebourg A., Cessot A., Pallier K., Danel C., Vacher-Lavenu M.-C., Laurent-Puig P., Terris B., Blons H. (2012). Histologic subtypes, immunohistochemistry, FISH or molecular screening for the accurate diagnosis of ALK-rearrangement in lung cancer: A comprehensive study of Caucasian non-smokers. Lung Cancer.

[80] Shaw A.T., Solomon B., Kenudson M.M. (2011). Crizotinib and testing for ALK. J. Natl. Compr. Canc. Netw..

[81] Mino-Kenudson M., Chirieac L.R., Law K., Hornick J.L., Lindeman N., Mark E.J., Cohen D.W., Johnson B.E., Jänne P.A., Iafrate A.J. (2010). A novel, highly sensitive antibody allows for the routine detection of ALK-rearranged lung adenocarcinomas by standard immunohistochemistry. Clin. Cancer Res..

[82] Paik J.H., Choe G., Kim H., Choe J.Y., Lee H.J., Lee C.T., Lee J.S., Jheon S., Chung J.H. (2011). Screening of anaplastic lymphoma kinase rearrangement by immunohistochemistry in non-small cell lung cancer: Correlation with fluorescence in situ hybridization. J. Thorac. Oncol..

[83] Wang R., Pan Y., Li C., Hu H., Zhang Y., Li H., Luo X., Zhang J., Fang Z., Li Y. (2012). The use of quantitative real-time reverse transcriptase PCR for 5’ and 3’ portions of ALK transcripts to detect ALK rearrangements in lung cancers. Clin. Cancer Res..

[84] Vanderlaan P.A., Yamaguchi N., Folch E., Boucher D.H., Kent M.S., Gangadharan S.P., Majid A., Goldstein M.A., Huberman M.S., Kocher O.N. (2014). Success and failure rates of tumor genotyping techniques in routine pathological samples with non-small-cell lung cancer. Lung Cancer.

[85] Sorber L., Zwaenepoel K., Deschoolmeester V., Van Schil P.E.Y., Van Meerbeeck J., Lardon F., Rolfo C., Pauwels P. (2017). Circulating cell-free nucleic acids and platelets as a liquid biopsy in the provision of personalized therapy for lung cancer patients. Lung Cancer.

[86] Bai Y., Zhao H. (2018). Liquid biopsy in tumors: Opportunities and challenges. Ann. Transl. Med..

[87] Wan J.C.M., Massie C., Garcia-Corbacho J., Mouliere F., Brenton J.D., Caldas C., Pacey S., Baird R., Rosenfeld N. (2017). Liquid biopsies come of age: Towards implementation of circulating tumour DNA. Nat. Rev. Cancer.

[88] Pantel K., Alix-Panabières C. (2019). Liquid biopsy and minimal residual disease—Latest advances and implications for cure. Nat. Rev. Clin. Oncol..

[89] Wang Y., Tian P.-W., Wang W.-Y., Wang K., Zhang Z., Chen B.-J., He Y.-Q., Li L., Liu H., Chuai S. (2016). Noninvasive genotyping and monitoring of anaplastic lymphoma kinase (ALK) rearranged non-small cell lung cancer by capture-based next-generation sequencing. Oncotarget.

[90] Hofman V., Bonnetaud C., Ilie M.I., Vielh P., Vignaud J.M., Fléjou J.F., Lantuejoul S., Piaton E., Mourad N., Butori C. (2011). Preoperative circulating tumor cell detection using the isolation by size of epithelial tumor cell method for patients with lung cancer is a new prognostic biomarker. Clin. Cancer Res..

[91] Ilie M., Hofman V., Long-Mira E., Selva E., Vignaud J.-M., Padovani B., Mouroux J., Marquette C.-H., Hofman P. (2014). “Sentinel” circulating tumor cells allow early diagnosis of lung cancer in patients with chronic obstructive pulmonary disease. PLoS ONE.

[92] Marquette C.-H., Boutros J., Benzaquen J., Ferreira M., Pastre J., Pison C., Padovani B., Bettayeb F., Fallet V., Guibert N. (2020). Circulating tumour cells as a potential biomarker for lung cancer screening: A prospective cohort study. Lancet. Respir. Med..

[93] Leroy S., Benzaquen J., Mazzetta A., Marchand-Adam S., Padovani B., Israel-Biet D., Pison C., Chanez P., Cadranel J., Mazières J. (2017). Circulating tumour cells as a potential screening tool for lung cancer (the AIR study): Protocol of a prospective multicentre cohort study in France. BMJ Open.

[94] Ilie M., Long E., Butori C., Hofman V., Coelle C., Mauro V., Zahaf K., Marquette C.H., Mouroux J., Paterlini-Bréchot P. (2012). ALK-gene rearrangement: A comparative analysis on circulating tumour cells and tumour tissue from patients with lung adenocarcinoma. Ann. Oncol..

[95] Pailler E., Adam J., Barthélémy A., Oulhen M., Auger N., Valent A., Borget I., Planchard D., Taylor M., André F. (2013). Detection of circulating tumor cells harboring a unique ALK rearrangement in ALK-positive non-small-cell lung cancer. J. Clin. Oncol..

[96] Tan C.L., Lim T.H., Lim T.K., Tan D.S.-W., Chua Y.W., Ang M.K., Pang B., Lim C.T., Takano A., Lim A.S.-T. (2016). Concordance of anaplastic lymphoma kinase (ALK) gene rearrangements between circulating tumor cells and tumor in non-small cell lung cancer. Oncotarget.

[97] Kulasinghe A., Lim Y., Kapeleris J., Warkiani M., O’Byrne K., Punyadeera C. (2020). The Use of Three-Dimensional DNA Fluorescent In Situ Hybridization (3D DNA FISH) for the Detection of Anaplastic Lymphoma Kinase (ALK) in Non-Small Cell Lung Cancer (NSCLC) Circulating Tumor Cells. Cells.

[98] Ilié M., Mazières J., Chamorey E., Heeke S., Benzaquen J., Thamphya B., Boutros J., Tiotiu A., Fayada J., Cadranel J. (2021). Prospective Multicenter Validation of the Detection of ALK Rearrangements of Circulating Tumor Cells for Noninvasive Longitudinal Management of Patients With Advanced NSCLC. J. Thorac. Oncol..

[99] Schwaederlé M.C., Patel S.P., Husain H., Ikeda M., Lanman R.B., Banks K.C., Talasaz A., Bazhenova L., Kurzrock R. (2017). Utility of Genomic Assessment of Blood-Derived Circulating Tumor DNA (ctDNA) in Patients with Advanced Lung Adenocarcinoma. Clin. Cancer Res..

[100] Aggarwal C., Thompson J.C., Black T.A., Katz S.I., Fan R., Yee S.S., Chien A.L., Evans T.L., Bauml J.M., Alley E.W. (2019). Clinical Implications of Plasma-Based Genotyping With the Delivery of Personalized Therapy in Metastatic Non-Small Cell Lung Cancer. JAMA Oncol..

[101] Leighl N.B., Page R.D., Raymond V.M., Daniel D.B., Divers S.G., Reckamp K.L., Villalona-Calero M.A., Dix D., Odegaard J.I., Lanman R.B. (2019). Clinical Utility of Comprehensive Cell-free DNA Analysis to Identify Genomic Biomarkers in Patients with Newly Diagnosed Metastatic Non-small Cell Lung Cancer. Clin. Cancer Res..

[102] Cui S., Zhang W., Xiong L., Pan F., Niu Y., Chu T., Wang H., Zhao Y., Jiang L. (2017). Use of capture-based next-generation sequencing to detect ALK fusion in plasma cell-free DNA of patients with non-small-cell lung cancer. Oncotarget.

[103] Dagogo-Jack I., Brannon A.R., Ferris L.A., Campbell C.D., Lin J.J., Schultz K.R., Ackil J., Stevens S., Dardaei L., Yoda S. (2018). Tracking the Evolution of Resistance to ALK Tyrosine Kinase Inhibitors through Longitudinal Analysis of Circulating Tumor DNA. JCO Precis. Oncol..

[104] Horn L., Whisenant J.G., Wakelee H., Reckamp K.L., Qiao H., Leal T.A., Du L., Hernandez J., Huang V., Blumenschein G.R. (2019). Monitoring Therapeutic Response and Resistance: Analysis of Circulating Tumor DNA in Patients With ALK+ Lung Cancer. J. Thorac. Oncol..

[105] Camidge D.R., Dziadziuszko R., Peters S., Mok T., Noe J., Nowicka M., Gadgeel S.M., Cheema P., Pavlakis N., de Marinis F. (2019). Updated Efficacy and Safety Data and Impact of the EML4-ALK Fusion Variant on the Efficacy of Alectinib in Untreated ALK-Positive Advanced Non-Small Cell Lung Cancer in the Global Phase III ALEX Study. J. Thorac..

[106] Li B.T., Janku F., Jung B., Hou C., Madwani K., Alden R., Razavi P., Reis-Filho J.S., Shen R., Isbell J.M. (2019). Ultra-deep next-generation sequencing of plasma cell-free DNA in patients with advanced lung cancers: Results from the Actionable Genome Consortium. Ann. Oncol..

[107] Dagogo-Jack I., Rooney M., Lin J.J., Nagy R.J., Yeap B.Y., Hubbeling H., Chin E., Ackil J., Farago A.F., Hata A.N. (2019). Treatment with Next-Generation ALK Inhibitors Fuels Plasma ALK Mutation Diversity. Clin. Cancer Res. Off. J. Am. Assoc. Cancer Res..

[108] Shaw A.T., Solomon B.J., Besse B., Bauer T.M., Lin C.C., Soo R.A., Riely G.J., Ignatius Ou S.H., Clancy J.S., Li S. (2019). ALK resistance mutations and efficacy of lorlatinib in advanced anaplastic lymphoma kinase-positive non–small-cell lung cancer. J. Clin. Oncol..

[109] Park C.-K., Kim J.-E., Kim M.-S., Kho B.-G., Park H.-Y., Kim T.-O., Shin H.-J., Cho H.-J., Choi Y.-D., Oh I.-J. (2019). Feasibility of liquid biopsy using plasma and platelets for detection of anaplastic lymphoma kinase rearrangements in non-small cell lung cancer. J. Cancer Res. Clin. Oncol..

[110] Nilsson R.J.A., Karachaliou N., Berenguer J., Gimenez-Capitan A., Schellen P., Teixido C., Tannous J., Kuiper J.L., Drees E., Grabowska M. (2016). Rearranged EML4-ALK fusion transcripts sequester in circulating blood platelets and enable blood-based crizotinib response monitoring in non-small-cell lung cancer. Oncotarget.

[111] Reclusa P., Laes J.-F., Malapelle U., Valentino A., Rocco D., Gil-Bazo I., Rolfo C. (2019). EML4-ALK translocation identification in RNA exosomal cargo ( ExoALK ) in NSCLC patients: A novel role for liquid biopsy. Transl. Cancer Res..

[112] Pailler E., Oulhen M., Borget I., Remon J., Ross K., Auger N., Billiot F., Ngo Camus M., Commo F., Lindsay C.R. (2017). Circulating Tumor Cells with Aberrant ALK Copy Number Predict Progression-Free Survival during Crizotinib Treatment in ALK-Rearranged Non-Small Cell Lung Cancer Patients. Cancer Res..

[113] Aieta M., Facchinetti A., De Faveri S., Manicone M., Tartarone A., Possidente L., Lerose R., Mambella G., Calderone G., Zamarchi R. (2016). Monitoring and Characterization of Circulating Tumor Cells (CTCs) in a Patient With EML4-ALK-Positive Non-Small Cell Lung Cancer (NSCLC). Clin. Lung Cancer.

[114] Oulhen M., Pawlikowska P., Tayoun T., Garonzi M., Buson G., Forcato C., Manaresi N., Aberlenc A., Mezquita L., Lecluse Y. (2021). Circulating tumor cell copy-number heterogeneity in ALK-rearranged non-small-cell lung cancer resistant to ALK inhibitors. NPJ Precis. Oncol..

[115] Manicone M., Scaini M.C., Rodriquenz M.G., Facchinetti A., Tartarone A., Aieta M., Zamarchi R., Rossi E. (2017). Liquid biopsy for monitoring anaplastic lymphoma kinase inhibitors in non-small cell lung cancer: Two cases compared. J. Thorac. Dis..

[116] Zhang Z., Shiratsuchi H., Palanisamy N., Nagrath S., Ramnath N. (2017). Expanded Circulating Tumor Cells from a Patient with ALK-Positive Lung Cancer Present with EML4-ALK Rearrangement Along with Resistance Mutation and Enable Drug Sensitivity Testing: A Case Study. J. Thorac. Oncol..

[117] Pailler E., Faugeroux V., Oulhen M., Mezquita L., Laporte M., Honoré A., Lecluse Y., Queffelec P., NgoCamus M., Nicotra C. (2019). Acquired Resistance Mutations to ALK Inhibitors Identified by Single Circulating Tumor Cell Sequencing in ALK-Rearranged Non-Small-Cell Lung Cancer. Clin. Cancer Res..

[118] Dietz S., Christopoulos P., Gu L., Volckmar A.-L., Endris V., Yuan Z., Ogrodnik S.J., Zemojtel T., Heussel C.-P., Schneider M.A. (2019). Serial liquid biopsies for detection of treatment failure and profiling of resistance mechanisms in KLC1-ALK-rearranged lung cancer. Cold Spring Harb. Mol. Case Stud..

[119] Sharma G.G., Cortinovis D., Agustoni F., Arosio G., Villa M., Cordani N., Bidoli P., Bisson W.H., Pagni F., Piazza R. (2019). A Compound L1196M/G1202R ALK Mutation in a Patient with ALK-Positive Lung Cancer with Acquired Resistance to Brigatinib Also Confers Primary Resistance to Lorlatinib. J. Thorac. Oncol..

[120] Sánchez-Herrero E., Blanco Clemente M., Calvo V., Provencio M., Romero A. (2020). Next-generation sequencing to dynamically detect mechanisms of resistance to ALK inhibitors in ALK-positive NSCLC patients: A case report. Transl. Lung Cancer Res..

[121] König D., Meier U.R., Klaeser B., Savic S., Pless M. (2020). Successful Treatment of a Resistant Subclone in ALK-Rearranged NSCLC. Case Rep. Oncol..

[122] Brinkmann K., Enderle D., Flinspach C., Meyer L., Skog J., Noerholm M. (2018). Exosome liquid biopsies of NSCLC patients for longitudinal monitoring of ALK fusions and resistance mutations. J. Clin. Oncol..

[123] Combaret V., Iacono I., Bellini A., Bréjon S., Bernard V., Marabelle A., Coze C., Pierron G., Lapouble E., Schleiermacher G. (2015). Detection of tumor ALK status in neuroblastoma patients using peripheral blood. Cancer Med..

[124] Chicard M., Colmet-Daage L., Clement N., Danzon A., Bohec M., Bernard V., Baulande S., Bellini A., Deveau P., Pierron G. (2018). Whole-Exome Sequencing of Cell-Free DNA Reveals Temporo-spatial Heterogeneity and Identifies Treatment-Resistant Clones in Neuroblastoma. Clin. Cancer Res..

[125] Cimmino F., Lasorsa V.A., Vetrella S., Iolascon A., Capasso M. (2020). A Targeted Gene Panel for Circulating Tumor DNA Sequencing in Neuroblastoma. Front. Oncol..

[126] Carneiro B.A., Pamarthy S., Shah A.N., Sagar V., Unno K., Han H., Yang X.J., Costa R.B., Nagy R.J., Lanman R.B. (2018). Anaplastic Lymphoma Kinase Mutation (ALK F1174C) in Small Cell Carcinoma of the Prostate and Molecular Response to Alectinib. Clin. Cancer Res..

[127] Siravegna G., Sartore-Bianchi A., Mussolin B., Cassingena A., Amatu A., Novara L., Buscarino M., Corti G., Crisafulli G., Bartolini A. (2017). Tracking a CAD-ALK gene rearrangement in urine and blood of a colorectal cancer patient treated with an ALK inhibitor. Ann. Oncol..

[128] Thompson J.C., Yee S.S., Troxel A.B., Savitch S.L., Fan R., Balli D., Lieberman D.B., Morrissette J.D., Evans T.L., Bauml J. (2016). Detection of Therapeutically Targetable Driver and Resistance Mutations in Lung Cancer Patients by Next-Generation Sequencing of Cell-Free Circulating Tumor DNA. Clin. Cancer Res..

[129] Mezquita L., Swalduz A., Jovelet C., Ortiz-Cuaran S., Howarth K., Planchard D., Avrillon V., Recondo G., Marteau S., Benitez J.C. (2020). Clinical Relevance of an Amplicon-Based Liquid Biopsy for Detecting ALK and ROS1 Fusion and Resistance Mutations in Patients With Non-Small-Cell Lung Cancer. JCO Precis. Oncol..

[130] Gadgeel S.M., Yan M., Paul S.M., Mathisen M., Mocci S., Assaf Z.J., Patel R., Sokol E.S., Mok T., Peters S. (2020). Blood first assay screening trial (BFAST) in patients (pts) with 1L NSCLC: ALK+ cohort updated biomarker analyses. In Proceedings of the ESMO. Ann. Oncol..

[131] Zhu Y., Jia R., Shao Y.W., Zhu L., Ou Q., Yu M., Wu X., Zhang Y. (2020). Durable Complete Response to Alectinib in a Lung Adenocarcinoma Patient With Brain Metastases and Low-Abundance EML4-ALK Variant in Liquid Biopsy: A Case Report. Front. Oncol..

[132] Kwon M., Ku B.M., Park S., Jung H.A., Sun J.-M., Lee S.-H., Ahn J.S., Park K., Ahn M.-J. (2020). Longitudinal monitoring by next generation sequencing of plasma cell-free DNA in ALK-rearranged non-small cell lung cancer (NSCLC) patients treated with ALK tyrosine kinase inhibitors. J. Clin. Oncol..

[133] Swalduz A., Ortiz-Cuaran S., Avrillon V., Marteau S., Martinez S., Clapisson G., Montane L., Pérol D., Green E., Howarth K. (2018). Fusion detection and longitudinal circulating tumor DNA (ctDNA) profiling in ALK+ non-small cell lung cancer (NSCLC) patients. J. Clin. Oncol..

[134] Yang Y., Huang J., Wang T., Zhou J., Zheng J., Feng J., Zhuang W., Chen J., Zhao J., Zhong W. (2020). Longitudinal circulating tumor DNA (ctDNA) analysis predicts response and reveals the resistance mechanisms of ensartinib in ALK+ NSCLC patients (pts) progressed on crizotinib: Updated analysis of a phase II clinical trial [abstract]. Cancer Res..

[135] Madsen A.T., Winther-Larsen A., McCulloch T., Meldgaard P., Sorensen B.S. (2020). Genomic Profiling of Circulating Tumor DNA Predicts Outcome and Demonstrates Tumor Evolution in ALK-Positive Non-Small Cell Lung Cancer Patients. Cancers.

[136] McCoach C.E., Blakely C.M., Banks K.C., Levy B., Chue B.M., Raymond V.M., Le A.T., Lee C.E., Diaz J., Waqar S.N. (2018). Clinical Utility of Cell-Free DNA for the Detection of ALK Fusions and Genomic Mechanisms of ALK Inhibitor Resistance in Non-Small Cell Lung Cancer. Clin. Cancer Res..

[137] Supplee J.G., Milan M.S.D., Lim L.P., Potts K.T., Sholl L.M., Oxnard G.R., Paweletz C.P. (2019). Sensitivity of next-generation sequencing assays detecting oncogenic fusions in plasma cell-free DNA. Lung Cancer.

[138] McCoach C.E., Le A.T., Gowan K., Jones K., Schubert L., Doak A., Estrada-Bernal A., Davies K.D., Merrick D.T., Bunn P.A. (2018). Resistance Mechanisms to Targeted Therapies in ROS1(+) and ALK(+) Non-small Cell Lung Cancer. Clin. Cancer Res..

[139] Gainor J.F., Dardaei L., Yoda S., Friboulet L., Leshchiner I., Katayama R., Dagogo-Jack I., Gadgeel S., Schultz K., Singh M. (2016). Molecular Mechanisms of Resistance to First- and Second-Generation ALK Inhibitors in ALK-Rearranged Lung Cancer. Cancer Discov..

[140] Noé J., Lovejoy A., Ou S.-H.I., Yaung S.J., Bordogna W., Klass D.M., Cummings C.A., Shaw A.T. (2020). ALK Mutation Status Before and After Alectinib Treatment in Locally Advanced or Metastatic ALK-Positive NSCLC: Pooled Analysis of Two Prospective Trials. J. Thorac. Oncol..

[141] Dietz S., Christopoulos P., Yuan Z., Angeles A.K., Gu L., Volckmar A.-L., Ogrodnik S.J., Janke F., Fratte C.D., Zemojtel T. (2020). Longitudinal therapy monitoring of ALK-positive lung cancer by combined copy number and targeted mutation profiling of cell-free DNA. EBioMedicine.

[142] Pös O., Biró O., Szemes T., Nagy B. (2018). Circulating cell-free nucleic acids: Characteristics and applications. Eur. J. Hum. Genet..

[143] Montani F., Marzi M.J., Dezi F., Dama E., Carletti R.M., Bonizzi G., Bertolotti R., Bellomi M., Rampinelli C., Maisonneuve P. (2015). miR-Test: A blood test for lung cancer early detection. J. Natl. Cancer Inst..

[144] Sozzi G., Boeri M., Rossi M., Verri C., Suatoni P., Bravi F., Roz L., Conte D., Grassi M., Sverzellati N. (2014). Clinical utility of a plasma-based miRNA signature classifier within computed tomography lung cancer screening: A correlative MILD trial study. J. Clin. Oncol..

[145] Ma J., Lin Y., Zhan M., Mann D.L., Stass S.A., Jiang F. (2015). Differential miRNA expressions in peripheral blood mononuclear cells for diagnosis of lung cancer. Lab. Investig..

[146] Li L.-L., Qu L.-L., Fu H.-J., Zheng X.-F., Tang C.-H., Li X.-Y., Chen J., Wang W.-X., Yang S.-X., Wang L. (2017). Circulating microRNAs as novel biomarkers of ALK-positive nonsmall cell lung cancer and predictors of response to crizotinib therapy. Oncotarget.

[147] Meng S., Zhou H., Feng Z., Xu Z., Tang Y., Li P., Wu M. (2017). CircRNA: Functions and properties of a novel potential biomarker for cancer. Mol. Cancer.

[148] Li Y., Zheng Q., Bao C., Li S., Guo W., Zhao J., Chen D., Gu J., He X., Huang S. (2015). Circular RNA is enriched and stable in exosomes: A promising biomarker for cancer diagnosis. Cell Res..

[149] Guarnerio J., Bezzi M., Jeong J.C., Paffenholz S.V., Berry K., Naldini M.M., Lo-Coco F., Tay Y., Beck A.H., Pandolfi P.P. (2016). Oncogenic Role of Fusion-circRNAs Derived from Cancer-Associated Chromosomal Translocations. Cell.

[150] Tan S., Gou Q., Pu W., Guo C., Yang Y., Wu K., Liu Y., Liu L., Wei Y.-Q., Peng Y. (2018). Circular RNA F-circEA produced from EML4-ALK fusion gene as a novel liquid biopsy biomarker for non-small cell lung cancer. Cell Res..

[151] Tan S., Sun D., Pu W., Gou Q., Guo C., Gong Y., Li J., Wei Y.-Q., Liu L., Zhao Y. (2018). Circular RNA F-circEA-2a derived from EML4-ALK fusion gene promotes cell migration and invasion in non-small cell lung cancer. Mol. Cancer.

[152] Calvo A.R., Ibarra G.H., Vibat C.R.T., Singh V.M. (2018). Detecting an ALK Rearrangement via Liquid Biopsy Enabled a Targeted Therapy-based Approach for Treating a Patient with Advanced Non-small Cell Lung Cancer. Oncol. Hematol. Rev..

[153] Wang Y., Yi J., Chen X., Zhang Y., Xu M., Yang Z. (2016). The regulation of cancer cell migration by lung cancer cell-derived exosomes through TGF-β and IL-10. Oncol. Lett..

[154] Webber J.P., Spary L.K., Sanders A.J., Chowdhury R., Jiang W.G., Steadman R., Wymant J., Jones A.T., Kynaston H., Mason M.D. (2015). Differentiation of tumour-promoting stromal myofibroblasts by cancer exosomes. Oncogene.

[155] Hoshino D., Kirkbride K.C., Costello K., Clark E.S., Sinha S., Grega-Larson N., Tyska M.J., Weaver A.M. (2013). Exosome secretion is enhanced by invadopodia and drives invasive behavior. Cell Rep..

[156] Vella L.J. (2014). The emerging role of exosomes in epithelial-mesenchymal-transition in cancer. Front. Oncol..

[157] Alderton G.K. (2012). Metastasis. Exosomes drive premetastatic niche formation. Nat. Rev. Cancer.

[158] Choi D.-Y., You S., Jung J.H., Lee J.C., Rho J.K., Lee K.Y., Freeman M.R., Kim K.P., Kim J. (2014). Extracellular vesicles shed from gefitinib-resistant nonsmall cell lung cancer regulate the tumor microenvironment. Proteomics.

[159] Li X.-Q., Liu J.-T., Fan L.-L., Liu Y., Cheng L., Wang F., Yu H.-Q., Gao J., Wei W., Wang H. (2016). Exosomes derived from gefitinib-treated EGFR-mutant lung cancer cells alter cisplatin sensitivity via up-regulating autophagy. Oncotarget.

[160] Brinkmann K., Enderle D., Koestler T., Bentink S., Emenegger J., Spiel A., Mueller R., O’Neill V., Skog J., Noerholm M. (2015). Abstract 545: Plasma-based diagnostics for detection of EML4-ALK fusion transcripts in NSCLC patients. Cancer Res..

[161] Combaret V., Audoynaud C., Iacono I., Favrot M.-C., Schell M., Bergeron C., Puisieux A. (2002). Circulating MYCN DNA as a tumor-specific marker in neuroblastoma patients. Cancer Res..

[162] Combaret V., Bergeron C., Noguera R., Iacono I., Puisieux A. (2005). Circulating MYCN DNA predicts MYCN-amplification in neuroblastoma. J. Clin. Oncol..

[163] Combaret V., Hogarty M.D., London W.B., McGrady P., Iacono I., Brejon S., Swerts K., Noguera R., Gross N., Rousseau R. (2009). Influence of neuroblastoma stage on serum-based detection of MYCN amplification. Pediatr. Blood Cancer.

[164] Kojima M., Hiyama E., Fukuba I., Yamaoka E., Ueda Y., Onitake Y., Kurihara S., Sueda T. (2013). Detection of MYCN amplification using blood plasma: Noninvasive therapy evaluation and prediction of prognosis in neuroblastoma. Pediatr. Surg. Int..

[165] Iehara T., Yagyu S., Gotoh T., Ouchi K., Yoshida H., Miyachi M., Kikuchi K., Sugimoto T., Hosoi H. (2019). A prospective evaluation of liquid biopsy for detecting MYCN amplification in neuroblastoma patients. Jpn. J. Clin. Oncol..

[166] Mosse Y.P., Laudenslager M., Longo L., Cole K.A., Wood A., Attiyeh E.F., Laquaglia M.J., Sennett R., Lynch J.E., Perri P. (2008). Identification of ALK as a major familial neuroblastoma predisposition gene. Nature.

[167] Bellini A., Pötschger U., Bernard V., Lapouble E., Baulande S., Ambros P.F., Auger N., Beiske K., Bernkopf M., Betts D.R. (2021). Frequency and Prognostic Impact of ALK Amplifications and Mutations in the European Neuroblastoma Study Group (SIOPEN) High-Risk Neuroblastoma Trial (HR-NBL1). J. Clin. Oncol..

[168] Schulte J.H., Lindner S., Bohrer A., Maurer J., De Preter K., Lefever S., Heukamp L., Schulte S., Molenaar J., Versteeg R. (2013). MYCN and ALKF1174L are sufficient to drive neuroblastoma development from neural crest progenitor cells. Oncogene.

[169] Lodrini M., Sprüssel A., Astrahantseff K., Tiburtius D., Konschak R., Lode H.N., Fischer M., Keilholz U., Eggert A., Deubzer H.E. (2017). Using droplet digital PCR to analyze MYCN and ALK copy number in plasma from patients with neuroblastoma. Oncotarget.

[170] Peitz C., Sprüssel A., Linke R.B., Astrahantseff K., Grimaldi M., Schmelz K., Toedling J., Schulte J.H., Fischer M., Messerschmidt C. (2020). Multiplexed Quantification of Four Neuroblastoma DNA Targets in a Single Droplet Digital PCR Reaction. J. Mol. Diagn..

[171] Kobayashi K., Mizuta S., Yamane N., Hamabata T., Maihara T., Usami I., Heike T. (2021). Cell-free DNA Oncogene Copy Number as a Surrogate Molecular Biomarker in ALK/MYCN-coamplified Neuroblastoma. J. Pediatr. Hematol. Oncol..

[172] Lan X., Bao H., Ge X., Cao J., Fan X., Zhang Q., Liu K., Zhang X., Tan Z., Zheng C. (2020). Genomic landscape of metastatic papillary thyroid carcinoma and novel biomarkers for predicting distant metastasis. Cancer Sci..

[173] Clifton K., Rich T.A., Parseghian C., Raymond V.M., Dasari A., Pereira A.A.L., Willis J., Loree J.M., Bauer T.M., Chae Y.K. (2019). Identification of Actionable Fusions as an Anti-EGFR Resistance Mechanism Using a Circulating Tumor DNA Assay. JCO Precis. Oncol..

[174] Bonvini P., Rossi E., Zin A., Manicone M., Vidotto R., Facchinetti A., Tombolan L., Affinita M.C., Santoro L., Zamarchi R. (2021). Case Report: Circulating Tumor Cells as a Response Biomarker in ALK-Positive Metastatic Inflammatory Myofibroblastic Tumor. Front. Pediatr..

